# A high-throughput 3D X-ray histology facility for biomedical research and preclinical applications

**DOI:** 10.12688/wellcomeopenres.19666.2

**Published:** 2023-12-08

**Authors:** Orestis L. Katsamenis, Philip J. Basford, Stephanie K. Robinson, Richard P. Boardman, Elena Konstantinopoulou, Peter M. Lackie, Anton Page, J. Arjuna Ratnayaka, Patricia M. Goggin, Gareth J. Thomas, Simon J. Cox, Ian Sinclair, Philipp Schneider

**Affiliations:** 1 μ-VIS X-ray Imaging Centre, Faculty of Engineering and Physical Sciences, University of Southampton, Southampton, England, SO17 1BJ, UK; 2 Institute for Life Sciences, University of Southampton, Southampton, UK; 3 Computational Engineering and Design, Faculty of Engineering and Physical Sciences,, University of Southampton, Southampton, England, SO17 1BJ, UK; 4 Clinical and Experimental Sciences, Faculty of Medicine, University of Southampton, Southampton, England, SO16 6YD, UK; 5 Biomedical Imaging Unit, Faculty of Medicine, University of Southampton, Southampton, England, SO16 6YD, UK; 6 University Hospital Southampton NHS Foundation Trust, Southampton, SO16 6YD, UK; 7 School of Cancer Sciences, Faculty of Medicine, University of Southampton, Southampton, England, SO16 6YD, UK; 8 High-Performance Vision Systems, Center for Vision, Automation & Control, AIT Austrian Institute of Technology, Vienna, Austria

**Keywords:** 3D X-ray Histology, XRH, Histology, microCT, μCT, μ-CT, virtual histology, 3D histology

## Abstract

**Background:**

The University of Southampton, in collaboration with the University Hospital Southampton (UHS) NHS Foundation Trust and industrial partners, has been at the forefront of developing three-dimensional (3D) imaging workflows using X-ray microfocus computed tomography (μCT) -based technology. This article presents the outcomes of these endeavours and highlights the distinctive characteristics of a μCT facility tailored explicitly for 3D X-ray Histology, with a primary focus on applications in biomedical research and preclinical and clinical studies.

**Methods:**

The UHS houses a unique 3D X-ray Histology (XRH) facility, offering a range of services to national and international clients. The facility employs specialised μCT equipment explicitly designed for histology applications, allowing whole-block XRH imaging of formalin-fixed and paraffin-embedded tissue specimens. It also enables correlative imaging by combining μCT imaging with other microscopy techniques, such as immunohistochemistry (IHC) and serial block-face scanning electron microscopy, as well as data visualisation, image quantification, and bespoke analysis.

**Results:**

Over the past seven years, the XRH facility has successfully completed over 120 projects in collaboration with researchers from 60 affiliations, resulting in numerous published manuscripts and conference proceedings. The facility has streamlined the μCT imaging process, improving productivity and enabling efficient acquisition of 3D datasets.

**Discussion & Conclusions:**

The 3D X-ray Histology (XRH) facility at UHS is a pioneering platform in the field of histology and biomedical imaging. To the best of our knowledge, it stands out as the world's first dedicated XRH facility, encompassing every aspect of the imaging process, from user support to data generation, analysis, training, archiving, and metadata generation. This article serves as a comprehensive guide for establishing similar XRH facilities, covering key aspects of facility setup and operation. Researchers and institutions interested in developing state-of-the-art histology and imaging facilities can utilise this resource to explore new frontiers in their research and discoveries.

## Introduction

**Figure 1.**
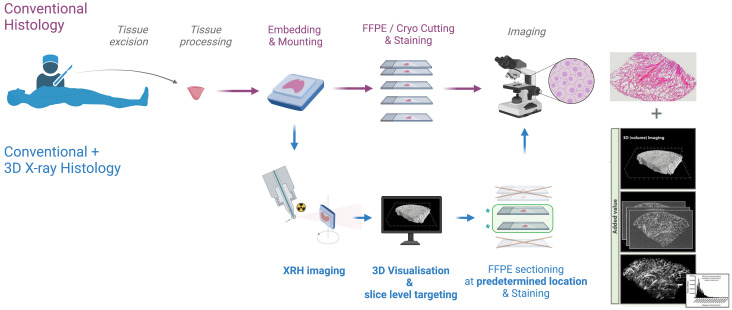
XRH integration into existing histology workflows. Non-destructive 3D XRH imaging can be seamlessly integrated into the protocols used for conventional 2D histology and enhance them by providing high-resolution 3D data. The XRH data can also be used to optimise physical sectioning of the tissue block for downstream conventional histology by identifying the areas of interest within the block and slicing accordingly.

3D X-ray Histology (XRH) is a microfocus computed tomography (microCT/μCT)-based technology that allows non-destructive, high-resolution three-dimensional (volumetric) imaging of tissue specimens for histology, with applications in the fields of biomedical research, pre-clinical investigations, and clinical histopathology. The term extends its applicability to encompass complementary X-ray μCT modalities, such as synchrotron-based μCT, phase-contrast imaging, photon-counting imaging, and more, reflecting its broader scope. In the context of this paper, 3D XRH represents a pioneering *attenuation μCT* -based technology developed as a result of the proof-of-principle study published in 2015 ^1^. In that study, we demonstrated that soft tissue samples routinely prepared for light microscopy-based conventional histology can be imaged using standard (attenuation-based) X-ray μCT. μCT achieves sufficient soft tissue contrast between the fixed soft tissue and the paraffin wax embedding medium for imaging the tissue microstructure in three dimensions (3D). This has been achieved by carefully selecting, adapting, and re-designing X-ray imaging hardware and optimising imaging protocols. With a spatial resolution at the μm scale, XRH offers detailed visualisation of microstructural features and supports correlative imaging workflows between other histology modalities across the scales such as 2D light microscopy-based histology ^2^, immunohistochemistry ^3^, but also electron microscopy ^4^. Importantly, it also enables imaging of routinely prepared tissue blocks, providing a comprehensive view of the specimen before any sectioning occurs. XRH provides various visualisation modes akin to those used in clinical radiology, including multi-planar reconstruction (MPR), maximum intensity projections (MIP), interactive volume rendering, and 'on-the-fly' arbitrary slicing ^2^. Beyond its imaging capabilities, XRH encompasses a complete and continually evolving workflow that ensures reliable sample traceability, enhances correlative imaging visualisation, supports data sharing and reporting, and automates metadata parsing. This makes it a valuable tool for advancing our understanding of tissue microstructure in the field of histology research and diagnostics. XRH ^2, 5^ offers a previously inaccessible view of soft tissue and its microanatomy for biomedical research, providing information about spatial heterogeneity ^6^, interconnectivity of components ^7^ and spatial growth (e.g. vascular invasion ^8^, tumour margin determination ^9^), which are not always accessible through 2D imaging such as slide-based microscopy for standard histology. Due to its spatial resolution at a microscopic scale, XRH is also well suited for microanatomic studies in preclinical and clinical applications across a wide range of disciplines, including pulmonology, urology, oncology, cardiology, orthopaedics, and neurology ^10^, as it is non-disruptive to the current histology workflows (see Figure 1). It is noted here that in this “introduction”, we do not provide a comprehensive literature review of all X-ray μCT-based techniques used and/or developed to date for studying soft tissue. Instead, we introduce the inception of our 3D X-ray Histology journey, along with the XRH facility and its rationale. Numerous colleagues have done extensive work in this field, and we refer readers to explore the domain through articles like ^10– 19^ for a more comprehensive overview.

The first complete system for 3D XRH using convectional (attenuation-based) μCT was developed by our team as part of a Wellcome Trust Pathfinder project (Development of micro-computed tomography (μCT) for enhanced diagnosis and prognosis in interstitial lung diseases (ILD), WT109682MA; 2016-17) in close collaboration with Nikon X-Tek Systems Ltd. The Med-X (prototype) 3D XRH system was optimised for soft tissue imaging in a biomedical research/clinical environment, deploying strategies to allow for a smaller footprint and lowering manufacturing cost ^2^; also see section 4.1. That system was commissioned in August 2016 and is installed in the Biomedical Imaging Unit (BIU) at the University Hospital Southampton (UHS) NHS Foundation Trust.

Follow-up funding from Wellcome Trust (‘Foundations for routine 3D X-ray histology’, 212940/Z/18/Z; 2019-23) allowed us to further develop a complete framework for non-destructive 3D (volume) imaging and analysis of standard formalin-fixed and paraffin-embedded (FFPE) tissue samples using µCT. This includes standardisation and automation of image acquisition and processing workflows, exploring new X-ray imaging hardware, and setting up a data and sample management infrastructure (software and hardware) that meets the tracking and reporting needs of a clinical workflow and easily links together images of the same tissue taken using different modalities. By using the experience gained with the Med-X system and other engineering scanners, two new anodes for the X-ray source, expanding the capabilities for both higher-resolution and higher throughput soft tissue imaging, have been designed, built and characterised. The data and sample management infrastructure has been the cornerstone of a new facility designed specifically for XRH ( www.xrayhistology.org), currently operating at the University Hospital Southampton (UHS) site. The XRH facility supports 3D histology imaging studies and provides services compatible with current clinical histology and research workflows. As it is jointly run by the μ-VIS X-ray Imaging Centre ( www.muvis.org) and the Biomedical Imaging Unit ( www.southampton.ac.uk/biu/), it brings together expertise in engineering and X-ray imaging with biomedical imaging, electron microscopy and histology.

In this paper, we present the 3D XRH facility in Southampton and how it meets the needs of the scientific community for biomedical research and (pre)clinical applications. We provide details on the specifications of the machines, explain how image data are acquired and processed and discuss general considerations relevant to setting up an XRH facility. As use cases of the technology, we put forward biomedical research and preclinical applications undertaken in different fields, and we outline our plans for future developments of the technology.

## 3D XRH facility design: Meeting community needs

**Figure 2.**
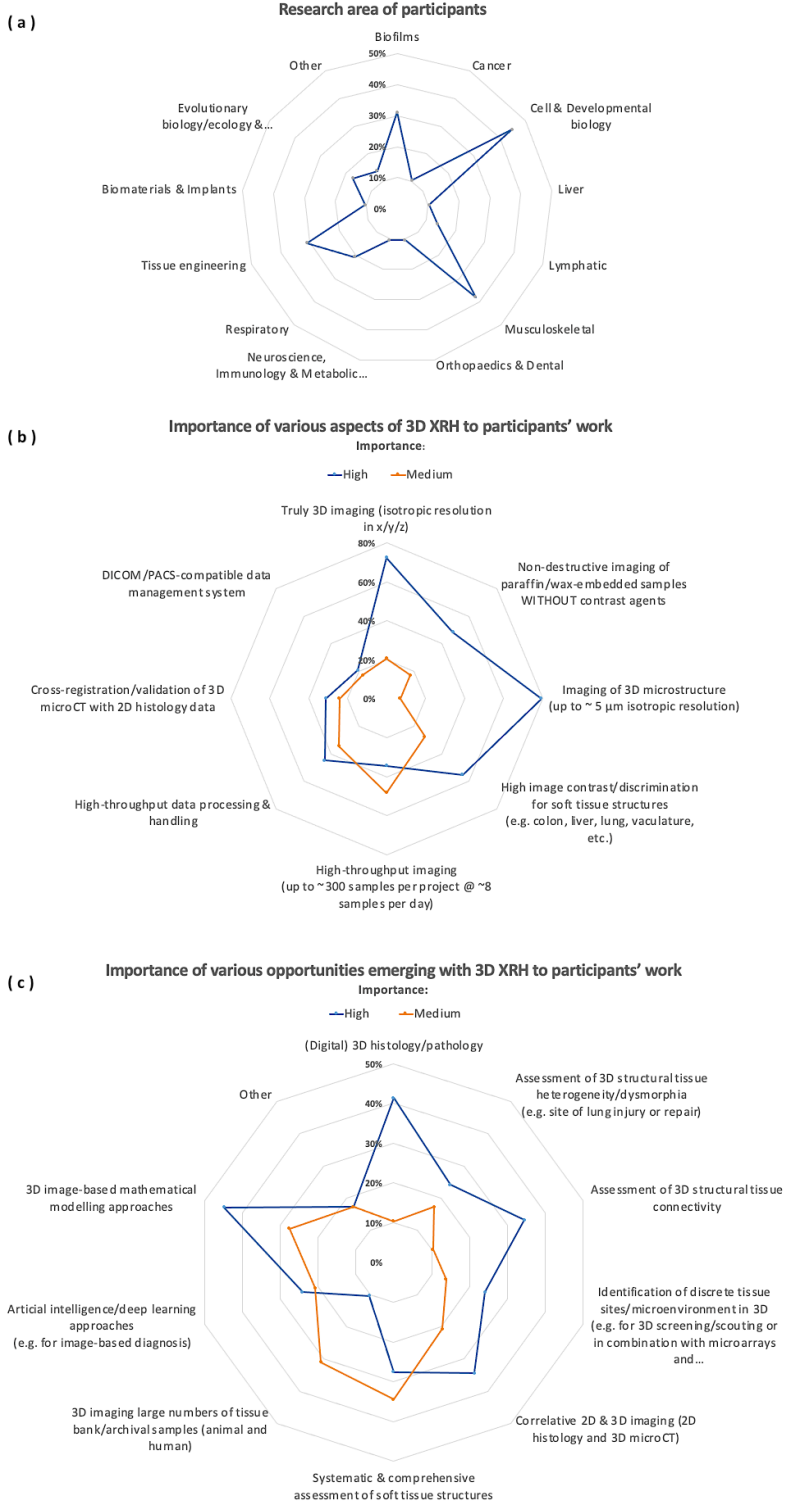
Summary of users’ survey. The survey collected information from both domestic (UK) and global collaborators and prospective users of the XRH facility, with the objective of comprehending the distinct requirements of the user community. The XRH facility was designed and tailored to cater to these demands; ( **a**): Research areas of Participants, ( **b**): Importance of various aspects of 3D XRH to participants’ work, ( **c**) Importance of various opportunities emerging with 3D XRH to participants’ work.

During the planning phase of the XRH facility, we reached out to our national (UK) and international network of collaborators and potential users of the XRH facility (51 biomedical and 15 non-biomedical). Using the questionnaire shown in the Supplementary Materials, we collected feedback about the aspects of the 3D XRH technology that would be most beneficial to their work and what new opportunities they could foresee accessing by exploiting XRH. 30 community members responded (response rate: 45%). The participants were working in the following fields: biofilms, cancer, cardiovascular, cell biology, developmental biology, liver, lymphatic system, musculoskeletal, neuroscience, respiratory, and tissue engineering research ( Figure 2a). Approximately 80% of the participants responded that imaging the microstructure in 3D using isotropic volume image elements (voxels) is highly important for their work. High image contrast from unstained tissues and high-throughput data processing and handling workflows were also highlighted as being of high importance by approximately half of the participants. Interestingly, high-throughput data processing and/or analysis seemed to rank higher in importance compared to high-throughput image acquisition, emphasising data processing/analysis as a critical aspect for the uptake of the technology. Other less popular aspects included the potential for correlative imaging between XRH and conventional histology, data compatibility with picture archiving and communication systems (PACS), and the DICOM digital image format ( Figure 2b). The community recognised that XRH raises meaningful opportunities for several applications. Almost 50% of the responders identified significant opportunities in image-based mathematical modelling and 3D histology for pathology applications and research. These opportunities were followed by applications in correlative imaging (between 3D XRH and conventional 2D histology data) and assessment of 3D structural element connectivity ( Figure 2c).

The results from this survey provided the rationale for the design of the XRH facility, starting from sample preparation up to processed outputs. A streamlined framework for the facility was needed to enable rapid sample throughput and image processing of high-resolution data without sacrificing the quality and resolution of 3D image acquisition (see Figure 1). Therefore, in addition to state-of-the-art hardware, software, and imaging protocols run by specialist experts, the IT infrastructure is a crucial aspect of the facility. It streamlines this workflow and makes data tracking future-proof. Below, we describe these key aspects of the facility and explain specific features included in the design to meet the community's needs.

## Microfocus tomography (μCT) imaging

**Figure 3.**
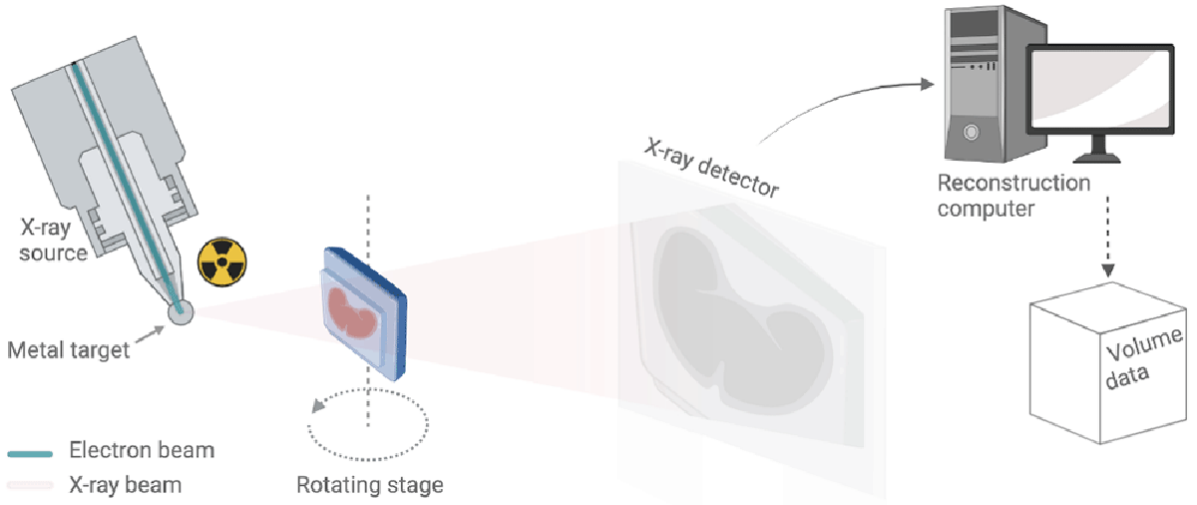
Schematic of a benchtop μCT scanner. The X-ray source on the left uses a beam of electrons fired at a metal target to produce a cone of X-rays. This conical X-ray beam then passes through the sample to the detector. The sample rotates on a rotation stage and for each angular step, a different X-ray radiography or projection image is taken and sent to the reconstruction computer for further processing (also see Supplementary Video 4).

In keeping with medical CT, μCT imaging is accomplished by placing the sample in the X-ray beam path and capturing projected X-ray absorption patterns (radiographs) over a large number of equidistant rotation angles (typically hundreds to thousands). In medical CT, high-resolution peripheral quantitative computed tomography (HR-pQCT) of extremities and the *in vivo* small animal scanners, the X-ray source and the detector rotate in a gantry system around the patient or the animal. In the laboratory μCT systems, however, the specimens (typically a few mm to cm in size) are scanned *ex vivo* with the X-ray source and detector fixed in space while the sample rotates on a rotating stage ( Figure 3). On completion of a scan, CT-specific algorithms are used to derive (reconstruct) the sample's internal structure. These algorithms use mathematical tools that combine the attenuation information captured on the radiographs to reconstruct the internal microstructure of the object by mapping local variability in attenuation properties, depended upon a number of factors including material- and electron-density and scattering and diffraction properties ^20^. These are then reconstructed into a 3D rectangular grid of cuboidal building blocks known as voxels. The principles of medical and µCT imaging and the various acquisition modes are comprehensively reviewed in 14, 20.

Both medical CT and conventional μCT use X-ray absorption (attenuation) for contrast. Here, we are focusing on the absorption-based technology. Still, for completeness, it is worth noting that X-rays also behave as waves (wave-particle duality), altering their wave properties like *phase* when interacting with matter. Phase alterations can be turned into intensity variations in the image ( *phase-contrast μCT)* using specialised hardware and appropriate reconstruction algorithms ^21, 22^. Phase imaging offers enhanced contrast compared to absorption techniques and has been used to visualise components of soft tissues, including kidney ^23^, peripheral airways ^24^, pulmonary and bronchial arteries ^25^, and muscle ^26^, it has therefore been identified as a promising approach for virtual histology ^27, 28^. However, most phase-contrast imaging techniques still rely on monochromatic or very narrow-band X-ray spectra accessible only in synchrotron radiation sources, which are few and far between. At the same time, laboratory-based phase-contrast imaging is gaining ground, with groups demonstrating the great potential of this approach in a more accessible setup. Limitations still remain, such as very long scan duration (>24h) ^28^, small field of view, low resolution ^29^ or complicated experimental setup with costly, X-ray-absorbing optical elements ^22^, which render these techniques impractical for use in a clinical setting. Nevertheless, phase-contrast images offer significant added value to conventional (absorption-based) μCT. Some of these drawbacks are slowly being addressed for specific histology applications, such as involved margins in breast specimens ^13^.

Depending on the image acquisition mode, reconstruction and/or processing method, voxels in 3D μCT images can be isotropic (equal size edge in *x*-, *y*-, and *z*-direction; x = y = z) or anisotropic (x ≠ y ≠ z ). Unless otherwise stated, voxels are considered isotropic by default, meeting the community requirements for truly 3D imaging ( Figure 2-B). Similar to key anatomical plane views in medical CT (axial, coronal, sagittal), the μCT data can also be viewed as a series of 2D slices, allowing data visualisation from different viewpoints.

## Results

### μCT systems at the 3D XRH facility

**Figure 4.**
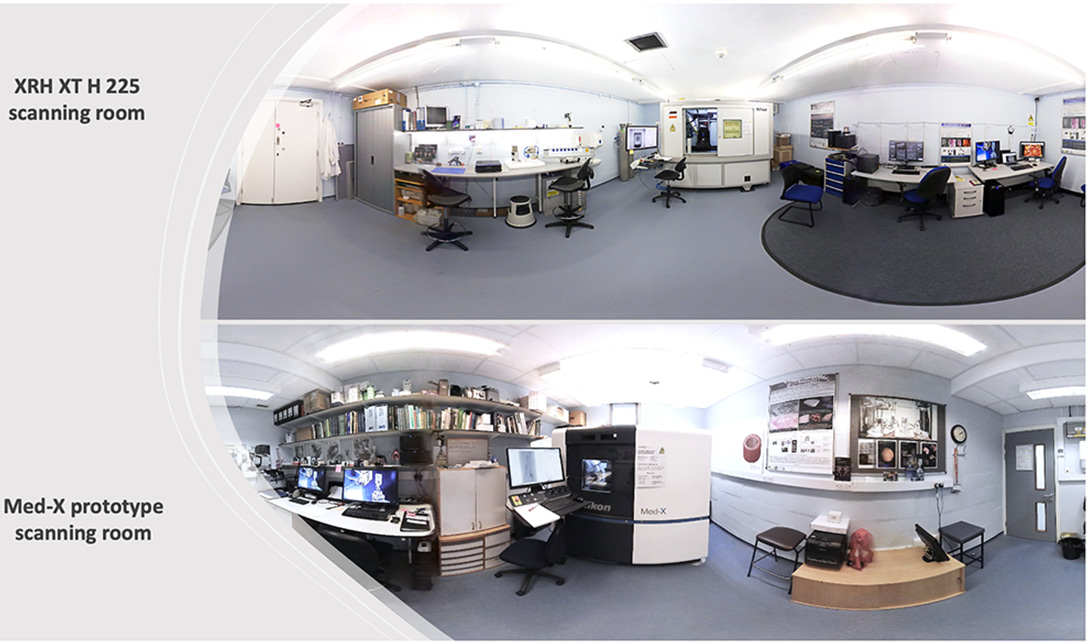
3D XRH facility at the University of Southampton. The facility is located at the University Hospital Southampton, and it is jointly run by the μ-VIS X-ray Imaging Centre and the Biomedical Imaging Unit. (Top) XRH XT H 225 scanning room. (Bottom) The Med-X prototype scanning room. Both μCT systems were custom-specified, designed, and built in a collaboration between the University of Southampton and Nikon X-Tek Systems Ltd (Tring, UK).

The μCT scanners of the 3D XRH facility at the University of Southampton ( Figure 4) are built using off-the-shelf hardware, such as commercially available flat-panel Perkin Elmer X-ray detectors, standard X-ray CT cabinets (XT H 225 series; Nikon X-Tek Systems Ltd, Tring, UK), and open-tube X-ray sources. The tube design results in a lower cost of ownership and increased flexibility in target assemblies. These standard parts have then been adapted to better suit soft tissue imaging.

***Med-X prototype system*.** The Med-X prototype system ( Figure 4) is the first commercial μCT scanner optimised for imaging standard FFPE specimens. These unstained soft-tissue specimens fit seamlessly into biomedical and clinical histopathology workflows. The system was specified on the back of the successful work ^1, 7^ conducted using a much larger walk-in experimental chamber, and the design was optimised for use in a medical/clinical environment. On this account, a lower energy X-ray source was used, allowing for a smaller footprint, lower weight, and reduced manufacturing cost. The system was built as part of a Wellcome Trust Pathfinder project in collaboration with Nikon X-Tek Systems Ltd and was commissioned in August 2016 at the University Hospital Southampton. 3DHISTECH Ltd has since acquired the technology and it is furthered developed into a commercially available scanner for histology applications under the name PANNORAMIC-X®.

The Med-X prototype is equipped with a 130 kVp/225 W multi-metal (W, Mo, Cu, Ag) microfocus reflection X-ray source with a focal spot size down to 2.5 μm, which allows μCT imaging at spatial resolutions down to approximately 5 μm (see extended data). Note that for the Med-X system at a magnification factor of 136x, the nominal minimum voxel size is 1.46 μm. However, what's critical is the source's spot size, which characterises the equipment and controls the maximum achievable spatial resolution. We've characterised all mentioned spot sizes using plain radiography and high-resolution phantoms such as Jima stars. Spatial resolution is conservatively assumed to be no better than 2x the spot size, as confirmed with a touching spheres phantom. The X-ray imaging detector is a 2000 × 2000 pixels panel with an active area of ca. 400 × 400 mm ^2^, which allows imaging specimens with an effective diameter less than 40 cm. Using this system at the 3D XRH facility, a range of soft tissues have been imaged to date, including healthy and fibrotic human lung FFPE samples ^2^, lymph node biopsies ^29^, human pulmonary lymphatics ^30^, placenta ^31^, HSC-3 cell-xenografted severe combined immunodeficiency (SCID) mouse tissue ^32^, bone marrow biopsies, as well as hard tissues ^33^ and micro-implants *in situ* such as cochlear implants ^34^.

***Customised XRH XT H 225 ST system*.** The XRH XT H 225 ST scanner ( Figure 4) is a customised μCT scanner based on an XT H 225 ST system (Nikon X-Tek Systems Ltd, Tring, UK). It is a next-generation XRH scanner for biomedical research, preclinical and clinical studies. Like the Med-X prototype, it has been custom-designed in house and optimised for imaging standard FFPE unstained soft tissue specimens, but sample throughput has been improved for clinically relevant turnaround times and scanner versatility increased as outlined below.

Specifically, the versatility has been increased by introducing an X-ray source (Nikon X-Tek Systems Ltd, Tring, UK) with interchangeable X-ray target heads, a bigger cabinet, and a dual detector geometry. X-ray generation can be achieved using any of the following targets:

(1) For **standard usage**: a 225 kVp/225 W multi-metal (tungsten (W), molybdenum (Mo), Copper (Cu), Silver (Ag) *reflection* target with a 2.5 μm focal spot size up to a power of 7 W
(2) For **high throughput**: a custom 130 kVp/130 W molybdenum *rotating* target with a 10 μm focal spot size up to a power of 50 W
(3) For **high resolution**: custom 180 kVp/20 W molybdenum *transmission* target with a 1.5 μm focal spot size up to a power of 5 W

Molybdenum has been shown to give better contrast when imaging at energies at or below 100kV, due to the “softer” X-ray spectrum produced; that is a spectrum exhibiting a lower mean energy compared to that of tungsten. This is mainly due to the large contribution of the lower energy K-shell peaks at 17 keV & 19 keV (compared to 60 keV & 67 keV for tungsten), which contribute approximately 30% to the total beam intensity at an acceleration voltage of 100 kVp ^35, 36^. Previously a molybdenum target has only been available as part of the multi-metal reflection target (1), which limits both the maximum power available and minimum focal spot size available. For the reflection target the maximum energy/power combination is only practical when using the tungsten option, on all other metals using this combination will result in the target melting. The integration of the innovative molybdenum rotating target, a pioneering feature of this facility, offers significant advantages over static reflection targets. This advancement allows for enhanced power generation and a smaller spot size, resulting in improved scanning capabilities. Particularly for applications that require high throughput at a spatial resolution lower than 20 µm, along with excellent soft tissue contrast, this technological enhancement proves to be highly beneficial ^37^. The rotation of the target wheel in a rotating target exposes different portions of the target material to the electron beam over time, resulting in improved heat dissipation. This allows the electron beam to maintain its optimal focus for higher X-ray power setting compared to a reflection target. The process of spot-defocusing, which spreads the heat load onto a larger area and safeguards the target from melting, also initiates at 14 W instead of 7 W. Furthermore, the defocusing curve is less steep compared to that of a reflection target, enabling the source to operate at a spot size smaller than 100 μm at 100 W. In practical terms, for scans with resolution requirements <10 μm this leads to shorter scan times for the same resolution or an increase in resolution by over a factor of 2x when using X-ray power exceeding 30W; that is a spatial resolution performance at 30 W of 45 μm and 90 μm for the rotated and the reflection target respectively. The molybdenum transmission target (3) is also a first of its kind target enabling higher resolution scans than has previously been possible using a molybdenum target in a Nikon lab-based CT system. This increased resolution comes at the cost of lower power, which is further compounded by the fact that the geometric magnification required to achieve this resolution means the detector has to be further away from the source, which further reduced the flux available per pixel due to the inverse square law. These additional targets expand the capabilities of the facility in ways not previously possible.

X-ray detection for volumetric imaging (μCT) or planar imaging (radiography) can be achieved with a 2850 × 2850 active pixel high-sensitivity flat panel detector, with an active area of 432 × 432 mm ^2^. The detector is mounted on a motorised gantry allowing additional flexibility with regards to source-to-detector distance, which in turn effects acquisition time through flux-modulation. The cabinet has also been adapted to accept a SANTIS 3204 HR photon-counting detector manufactured by DECTRIS ^38^ for noise-free (no readout noise and dark current) and energy-sensitive X-ray detection. This detector has a resolution of 4150 × 514 pixels and allows for *direct detection* of X-rays rather than needing a scintillator and optical detection system ^39, 40^. The use of direct photon-counting techniques within XRH is currently being investigated.

**Figure 5.**
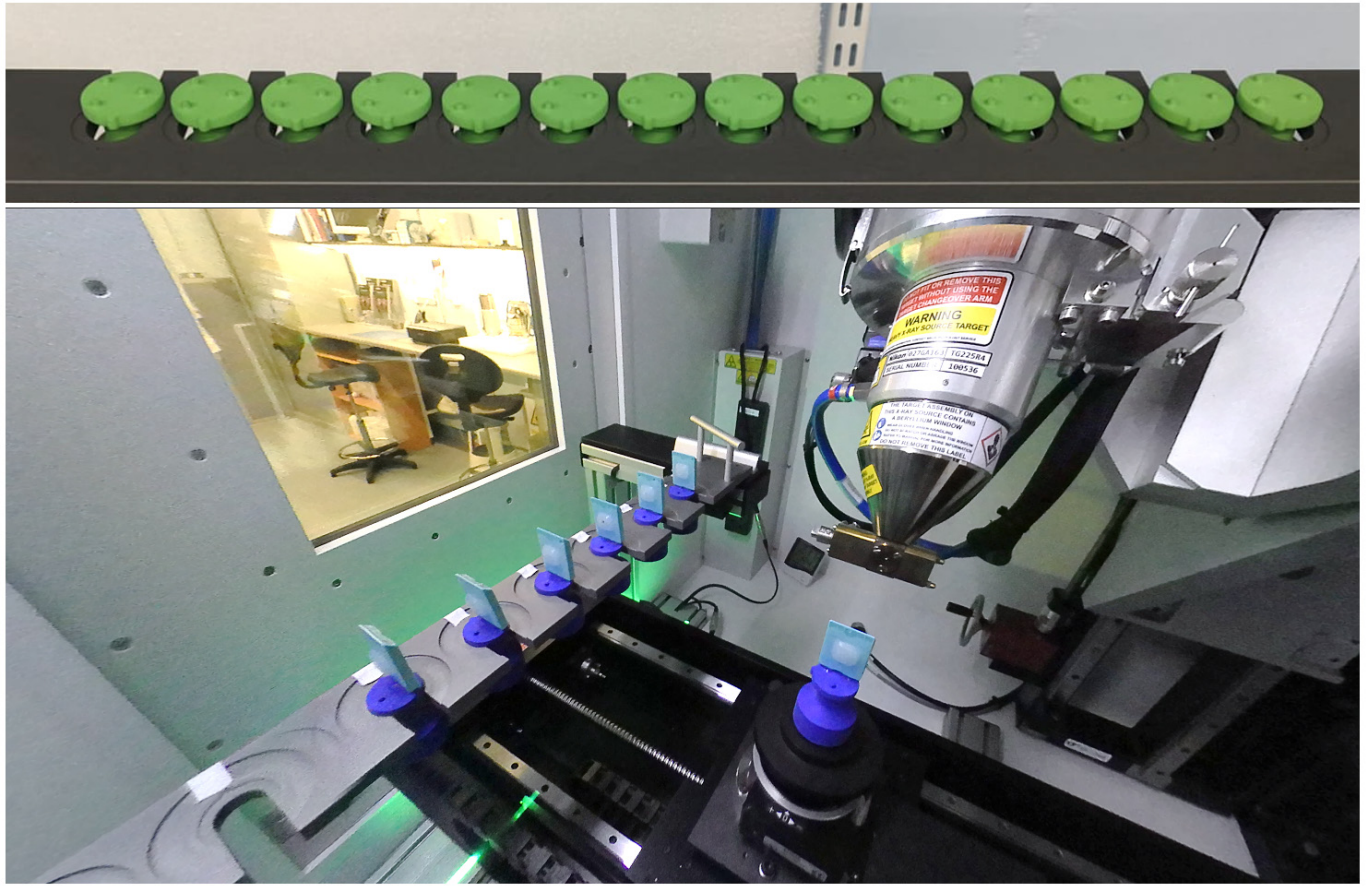
The sample exchange autoloader system utilized for uninterrupted micro-computed tomography (μCT) scanning at the University of Southampton's 3D X-ray Histology (XRH) facility. The top panel shows a 14-slot rack capable of holding samples with a 30 mm diameter per slot, while the bottom panel displays a 10-slot rack with dual slots accommodating samples with 30 mm and 60 mm diameters. Seven conventional histology cassettes are loaded in the bottom rack for scanning.

The XRH XT H 225 ST scanner is equipped with a sample exchange autoloader ( Figure 5 & video ^41^) which allows the system to automatically process a batch of samples. The autoloader system can either take up to 10 samples as a combination of large-format and standard histology cassettes. It can also support a rack of 14 standard cassettes. The time taken to exchange the samples allows the automatic reconstruction system to process scans in the batch while the batch is progressing. These features combined enable the scanner to run continuously overnight and over several-days, dramatically increasing the sample throughput available. The later directly impacts the cost of imaging, significantly reducing the cost per scan by making a more effective use of the otherwise idling time over-night and by reducing operators’ time. For example, a batch scan of multiple 2h-long scans without the autoloader would require operators’ intervention every two hours and will result in a throughout of up to 5 samples per (working) day, when the autoloader requires an upfront operator’s intervention to program the batch (1–2 hours) and resulting throughput increases to 11 samples by utilising overnight imaging. The cost-per-scan then decreases by a factor of ~ 2.5 when the autoloader is used. This key feature has enabled an increase in throughput of XRH samples and enabled the lab to continue to operate during the COVID-19 pandemic scanning throughout the week but only requiring access one day a week to setup the autoloader with multiple scans.

The autoloader system eliminates the need for a separate robot system to exchange samples on the scanner manipulator. Instead, it utilizes the existing manipulator by replacing the flat plate of the rotate stage with a tapered rod. This tapered rod fits into a matching receptacle in the autoloader sample holders, which are located in milled recesses within slots of a rack. The rack is equipped with micro-switches to detect collisions, pausing the process when a collision occurs until an operator intervenes. When not in use, as is the case when the SANTIS is fitted, the rack mounting system can be removed or fitted with blanks that satisfy the interlocked micro-switches. The sample holders are 3D printed, initially using an SLS printer and later developed in-house using FDM and resin printers for prototyping and high accuracy printing respectively. The flexibility of having both printers on-site enables rapid design, testing, and production of new mounts. The software control of the autoloader system integrates with the scanner's Inspect-X control system through inter-process communication (IPC). The setup involves determining scan parameters and archiving a protocol in Inspect-X. Samples are affixed to the holders using hot-melt glue or cyanoacrylate, allowing multiple scans per sample with the option to change parameters between scans. Changing the target metal requires a physical adjustment, meaning all scans on a tray must use the same target material. Trays can be configured on a separate computer and started later. The IPC also facilitates a utility for on-demand pickup or return of individual samples.

The improvements to the X-ray scanning hardware that have been integrated into both the Med-X and XRH scanners including customised X-ray anodes, auto-loader and ability to mount a DECTRIS SANTIS 3204HR, enrich the user experience by providing a singular, comprehensive suite of cutting-edge equipment specifically tunned for soft-tissue imaging available at a single facility. This resource empowers users with an expanded and diverse range of acquisition modes to choose from.

***Innovative Hardware Advancements*.** The substantial portion of this project has been dedicated to improving hardware components. In collaboration with our industrial partner, we have focused on optimising our systems with a primary objective of achieving the highest possible image quality when imaging standard soft-tissue FFPE tissue cassette specimens. These advancements have been designed with practical clinical applications in mind. We have focused on several key modifications to our system including:

- Adjusting the electron-beam angle of incidence onto the target to mitigate the heel-effect, which, if unaddressed, results in an uneven mean energy distribution of the X-ray beam from the bottom to the top of the illumination field. This adjustment is crucial for precise quantitative imaging and ensuring consistent tissue visualisation across the entire illuminated area.
- Modifying the X-ray source housing to diminish the generation of secondary X-ray sources arising from interactions between back-scattered electrons and the target housing. Although this occurrence poses minimal concerns when imaging materials with high attenuation contrast, it presented challenges when imaging unstained FFPE specimens. These specimens require us to operate within extremely narrow contrast windows within which "phantom" images of the specimens, stemming from the secondary sources, became noticeable.
- Co-designing the Med-X prototype enclosure to reduce its footprint and its weight by lowering the system's maximum energy to 130 kVp using a soft-limited 160 kVp source. We also made adjustments to its internal components by adding cover-panels, to facilitate hassle-free cleaning and diminishing the potential for biohazardous contaminations in clinical settings.
- Modifying the standard tungsten rotating target to a molybdenum rotating target, to allow for higher flux soft X-ray imaging and improving throughput within more clinically relevant time-frames. This was paired with the development and deployment of our XRH XT H 225 ST system of our automatic sample exchanger. This upgrade was complemented by the development and implementation of our automatic sample exchanger in the XRH XT H 225 ST system.
- Specifically designing the sample racks for the autoloader system. With that we have ensured their adaptability to accommodate either the maximum number of standard 30 mm histology cassettes or to offer 10 slots capable of holding either a standard 30 mm cassette or 60 mm giga-cassettes.
- Modifying the standard tungsten transmission target to a molybdenum transmission target to allow imaging with softer X-rays at much higher resolution.

### Data visualisation and processing

Following μCT imaging, several visualisation options are made available to the users as part of the standard image post processing workflows at the 3D X-ray facility. As in radiology, μCT images and hence XRH data can be presented to the viewer using a range of 2D and 3D visualisation modes which include, but are not limited to, multi-planar reconstruction, maximum intensity projections, or interactive volume rendering. Depending on the nature of the visualisation, the renderings are offered as single volume files (including: .raw, .tif, .DICOM), which can be opened with any available 2D/3D image viewer (e.g., Fiji/ImageJ ^42^) and complemented with video files when motion or scrolling is important for 3D data interpretation. These video files are significantly smaller than the reconstructed volume and can be easily and quickly sent to collaborators as part of an initial report about the scan. This allows users to review their data with minimal effort and without requiring prior experience with visualisation software. The generation of these videos has been semi-automated by the creation of a toolbox of Fiji macros, which is made available to readers through Zenodo.org ( https://doi.org/10.5281/zenodo.8253271).

**Figure 6.**
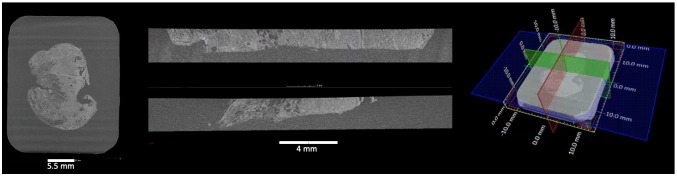
Multiplanar 2D viewing of 3D XRH data. (Left) The xy-plane is defined by convention as the plane parallel to the histology cassette, where one xy-slice (blue plane on the localiser shown on the right) is shown here. (Middle) One yz-slice at the top (red on the localiser shown on the right) and one xz-slice at the bottom (green on the localiser sown on the right) that have been selected. They are orthogonal to each other and to the xy-plane. (Right) Localiser that combines one xy-slice and the position of the other ortho-planes (yz-slice: red; xz-plane: green). Using ortho-plane viewing, the user can scroll through the depth, width, and height of the specimen, zoom, pan, and perform dimensional measurements.

***2D visualisation*.** A single slice is a cross-sectional (2D) view of the 3D image stack along a selected plane. XRH data sets are normally oriented (resliced) in a way that scrolling through the stack along the *xy*-plane emulates the physical histology slicing of the tissue block. This way, every *xy*-slice through the image stack (blue plane in Figure 6) is parallel to the histology cassette (where applicable) and the *xy*-slice scrolling runs from the wax block's surface towards the cassette (Supplementary Video 1), similarly to action of the microtome knife during physical sectioning in standard histology workflows. Orthogonal *xy*-, *xz-*, and *yz*-planes (blue, red, and green planes in Figure 6, Supplementary Video 2, and Supplementary Video 3) are also referred to as ortho-planes, which can be visualised in parallel for multiplanar viewing (see Figure 6), similar to visualisation of medical CT or magnetic resonance imaging (MRI) data. X-ray μCT slices are single channel images, which usually represent the intensity value of the captured signal (X-ray attenuation). Although μCT slices are greyscale, pseudo colouring can be applied through look-up tables (LUT), where each pixel’s grey value is associated with a colour. This can be used to generate photorealistic renderings for communicating the data with a wider audience, or to “digitally stain” the μCT image to emulate conventional histology outputs ^9^.

**Figure 7.**
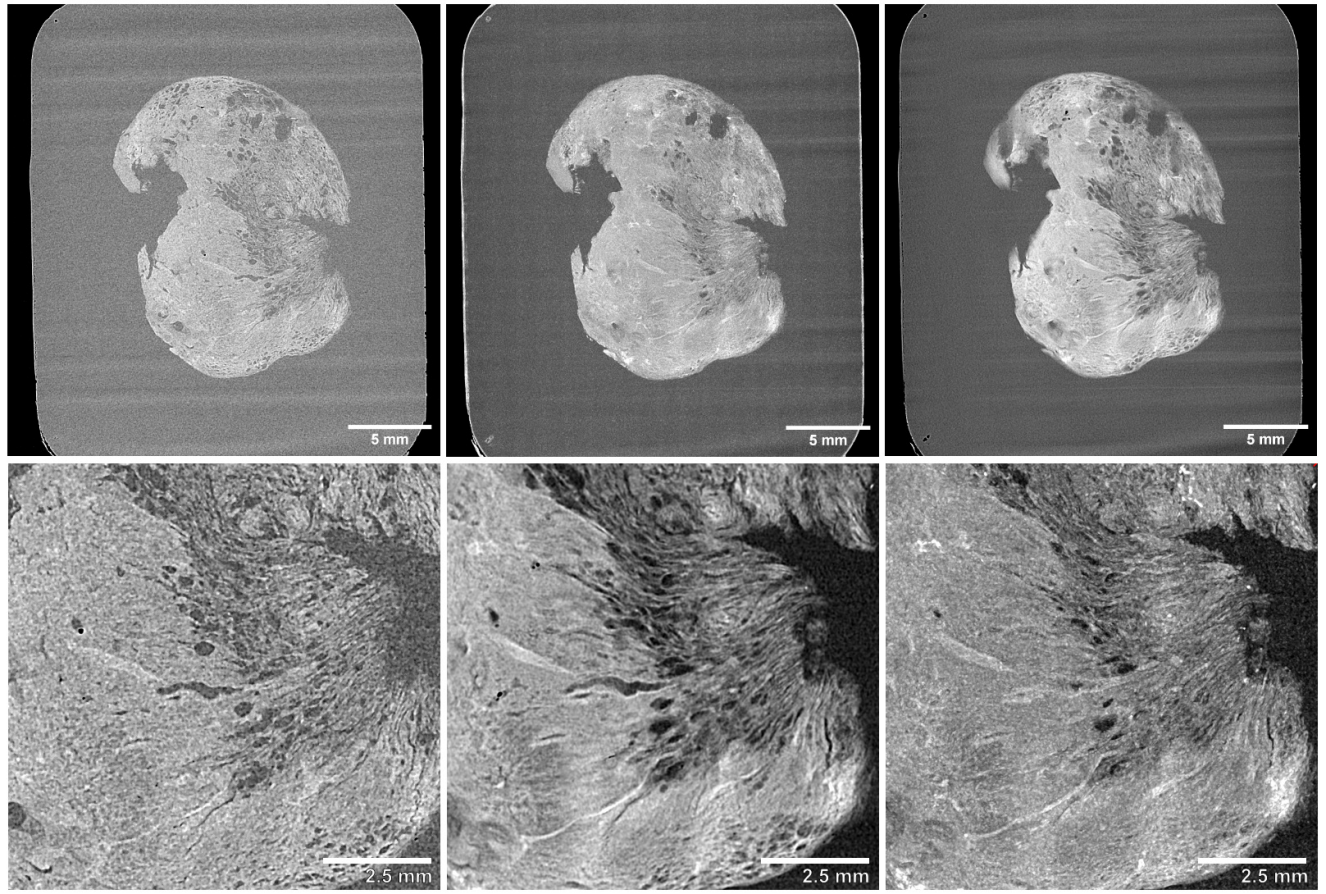
Thick-slice 2.5D viewing of 3D XRH data. Top row shows a whole-block XRH image of a head and neck solid tumour specimen, while the bottom row displays a 10 x 10 mm ^2^ detail view of the lower-right area of the tissue. (Left) Single slice view, (Middle) 20-slice average intensity projection “AVG”, (Right) 20-slice maximum intensity projection “MIP”.

***2.5D (thick slice modes) visualisation*.** Thick-slice viewing is a viewing mode that allows rendering of the voxel intensity of multiple consecutive slices within a 3D data set onto a single slice, based on specific criteria or operations ( Figure 7). A thick-slice image represents the result of these criteria or operations. For instance, a thick-slice view can be generated by applying one of the following projection methods across a pre-selected number of slices ( *n*), which defines the slice thickness:

(1) Maximum intensity projection (MIP): A MIP analyses each voxel at a specific position ( *X*, *Y*) within the *xy*-plane, for *n* slices (e.g. ( *X*, *Y*,1), ( *X*,Y,2), ... , ( *X*, *Y*, *n*)) along the *z*-axis, and renders the voxel onto the viewing plane, which exhibits the maximum value ( Figure 7 and Supplementary Video 5).
(2) Average intensity projection (AVG): an AVG analyses each voxel at a specific position ( *X*, *Y*) within the *xy*-plane across n slices (e.g. ( *X*, *Y*,1), ( *X*,Y,2), ... , ( *X*, *Y*, *n*)) along the *z*-axis and renders the average value of those voxels onto the viewing plane ( Figure 7 and Supplementary Video 6).
(3) Sum intensity projection (SUM): SUM analyses each voxel at a specific position ( *X*, *Y*) within the *xy*-plane across n slices (e.g. ( *X*, *Y*,1), ( *X*,Y,2), ... , ( *X*, *Y*, *n*)) along the *z*-axis and renders the sum of the value of those voxels onto the viewing plane ( Figure 7 and Supplementary Video 7).

Thick-slice modes are particularly useful when looking at large anatomical structures that extend outside the 2D plane. By creating thicker slices, the observer can get a better view of the overall structure which is particularly useful in visualisation the development of vessels and airways, tumour infiltration, emphysema, microcalcifications or thrombi.

*Thick-slice rolling* is a 2D thick-slice viewing that allows rolling of a pre-selected number of slices ( *n*) along the *z*-axis of the 3D data. A single thick-slice roll forwards is accomplished by translating the thick-slice by one single slice forwards; that is moving forward by one (+1) slice from the first and *n ^th^* element and reapplying the criteria or operations to the new slice sub-stack *.* The process is schematically outlined below for a slice stack of N slices, a thick slice thickness of n=5 and operation of “average intensity”:

Image stack (slice #): [ 1,2,3,4,5,6 …N _stack_ ]
Image stack of 5-slice AVG _thick-slice-1_: [ | **1,2,3,4,5**| ^AVG^,6, … N _thick-stack_ ]
Image stack of 5-slice AVG _thick-slice-2_: [ -,| **2,3,4,5,6**| ^AVG^,7, … N _thick-stack_ ]
Image stack of 5-slice AVG _thick-slice-5_: [ -,-,-,-,| **5,6,7,8,9**| ^AVG^,10, … N _thick-stack_ ]

As with single thick-slices, “thick-slice rolling” allows insights into the 3D shape of complex structures, that are difficult to be interpreted in 2D, and they do so in a dynamic way allowing the viewer to scroll in and out of plane and follow the development of that structure in the volume. Examples of these modes can be seen in Video 5, Video 6 and Video 7.

**Figure 8.**
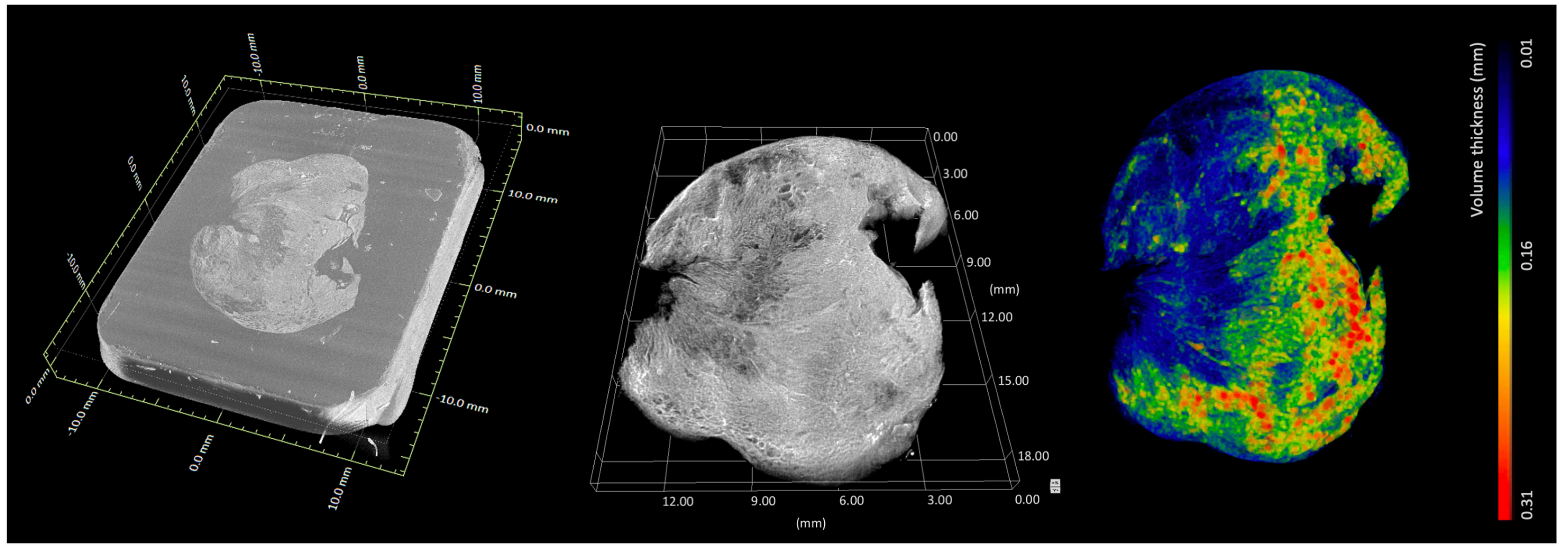
Whole-block XRH imaging of a conventionally prepared FFPE sample. Histology cassette not rendered for clarity. (Left) Volumetric rendering showing the tissue sample inside the wax (embedding medium of the sample). (Middle) volumetric rendering of the tissue. (Right) volumetric rendering of local 3D tissue thickness, where the colour bar ranges from 0.01 mm (blue) up to 0.31 mm (red). Images generated using Dragonfly (Object Research Systems).

***3D (volume) visualisation*.** Volumetric rendering is a 3D visualisation mode used for displaying the 3D grid of voxels contained in 3D data sets such as XRH data sets ( Figure 8). Volume rendering algorithms visualise images of volumetric data sets by mapping data values to opacity and colour, without explicitly extracting geometric surfaces of the object. Each voxel corresponds to a location in data space and has one or more data values associated with it. In the case of a CT reconstruction, volumetric rendering visualises the 3D map of appropriately scaled X-ray attenuation values or more precisely, linear X-ray attenuation coefficients. Roughly speaking, these values represent the density distribution within the sample, which in turn provides information about the microanatomy of the tissue in 3D. Whole-sample visualisation allows for a multi-perspective examination of the tissue, minimizing the risk of inadequate tissue sampling associated with destructive conventional histology, where slides are cut once by a microtome in a pre-defined plane. This has been tested by a team at the Department of Pathology at the Memorial Sloan Kettering Cancer (New York, USA), one of the very early adopters of XRH, where XRH-guided intraoperative cryo-sectioning was used on the sites of lesions and surgical margins ^9^. In line with the 2D thick-slice renderings, 3D MIP or AVG rotation can also be generated. In this mode, the dataset is rotated about a user-defined axis, and the rotation allows for depth and perspective interpretation of the brightest features as demonstrated previously (see Supplemental Video in 6). 3D renderings are also used to visualize novel quantitative features extracted from the volumetric data such the local 3D thickness ^6^ (see also Figure 8).

***Image processing and quantitative analysis*.** One of the primary needs for users of the 3D XRH facility is efficient data processing and handling workflows (as outlined previously), including the quick extraction of data from generated images. To address this, the XRH team offer their expertise in a variety of software suites available on-site for processing and analysing µCT datasets. The software options range from open-source suites, such as Fiji/ImageJ ^42^, ITK Snap ^43^ or HOROS® (The Horos Project) to commercial software suites such as VGSTUDIO MAX (Volume Graphics), Avizo® (Thermo Fisher Scientific), Simpleware (Synopsys Inc), OsyriX® (Pixmeo), or Dragonfly (Comet Technologies Canada Inc.). Additionally, designated high-powered computers and a custom visualisation suite with enterprise grade GPUs are available for use by facility users along with access to the University of Southampton's high-performance computing facility, IRIDIS 5. On-site training courses and one-on-one problem-solving sessions are also offered to facility users.

A bottleneck in speed of data extraction is often in the segmentation, or digital isolation, of specific features in the µCT data set. Segmentation is necessary to undertake quantitative analysis of these features. To segment an image through automated processes, high image quality is vital. On this account, µCT scanning protocols have been optimised for soft tissues over the last few years, in terms of signal-to-noise ratio, image contrast and other image quality factors. Moreover, image post processing routines have been streamlined including processing scripts, for tasks such as image enhancement, normalisation, or artefact reduction that can be run automatically on open-source software such as Fiji/ImageJ. This is offered to users, permitting high-throughput comparative analysis on multiple µCT datasets, which was another key requirement to be met for the 3D X-ray histology facility. Case study 1, presented below, is a demonstration of the use of imaging processing tools and the quantitative nature of the µCT data.

***Remote visualisation*.** There are multiple ways of providing remote visualisation: using display forwarding techniques such as Remote Desktop which is discussed further in this section, as well as web based viewing options which are discussed in Section 8. Options for sharing the data are discussed a later section. During the COVID-19 pandemic remote visualization has proven valuable for internal users at the University of Southampton as this it provided access to the computational power and storage needed to process the data. This was limited to internal users because opening the infrastructure wider would require consideration of: different data policy models, cybersecurity measures for data protection, establishing user accounts and institutional policy adherence, managing licensing issues for commercial software, and safeguarding intellectual property. By carefully evaluating these factors, remote visualization can be effectively implemented for first-time users, offering them significant advantages while ensuring data security, policy compliance, and addressing licensing and IP concerns. Different methods for sharing the datasets currently in use are discussed in section 6.2, and future options to be investigated are covered in section 8.

**Figure 9.**
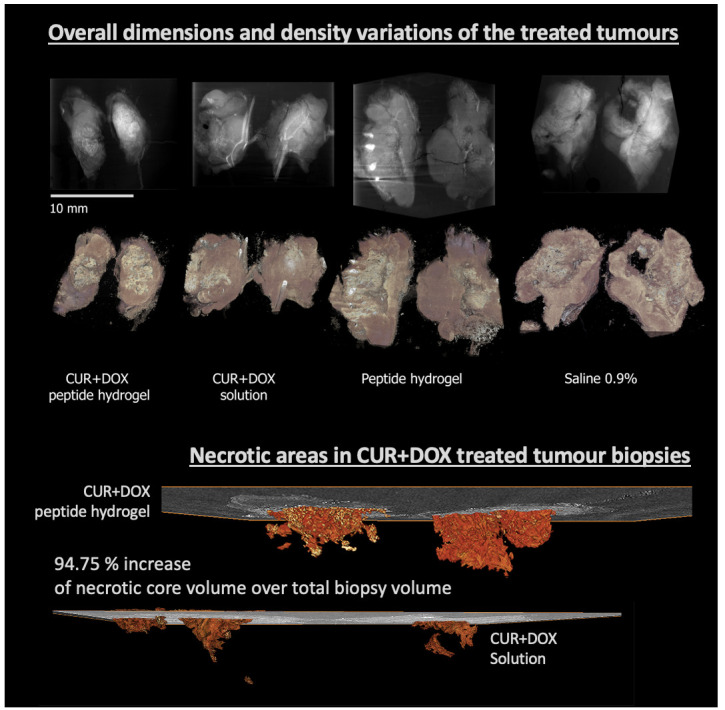
Quantitative imaging for cancer research. (Top section) Average intensity projection (AvgIP) and 3D volume rendering of the tumours studied in 25; (Bottom section): 3D (volume) rendering of the segmented ‘necrotic core’ of two tumours treated with two different formulation, showing the extent and the morphology of the necrotic core in space, rendered along with a representative central 2D μCT slice. Quantitative analysis allowed to accurately access the volume of the necrotic core (29.83 mm ^3^ vs. 13.16 mm ^3^) as well as the core to total tumour ratio: 2.58% of the tumour volume in the DOX+CUR solution-treated tumour was necrotic compared to 5.29% on the DOX+CUR peptide hydrogel-treated one.

***Case study 1: Quantitative imaging for cancer research/diagnosis*.** In this study ^32^, XRH was used to assess the synergistic anti-tumour potency of novel drug formulations. XRH was employed to provide high-resolution, 3D (volumetric) microanatomical information of tumour response to treatment by analysing the phenotype of the excised tumours ( Figure 9). XRH imaging of the excised tumours allowed us to quantitatively analyse the volumetric characteristics of anatomical features, such as total tumour volume, its necrotic core, and calcifications of tumours treated with different formulations. Measuring the ratio between the total tumour volume and the necrotic tissue volume highlighted the efficacy of the Doxorubicin and Curcumin (DOX+CUR) peptide hydrogels in enhancing tumour necrosis and suppressing cell proliferation. In summary, we could show that XRH is a powerful tool for providing quantitative high-resolution anatomical information of tumour biopsies after intra tumoral administration of chemotherapeutic formulations ^32^.

***Bespoke data visualisation: Correlative imaging and image-based mathematical modelling*.** Bespoke visualisation options, such as correlative imaging renderings or modelling-based visualisation are important to interrogate XRH data in terms the underlying function of the biological features studied. These are application-specific modes that need to be optimised on a project-by-project basis. On our users’ survey 34% of responders indicated that 2D or 3D correlative imaging could be of interest in the future and 50% were interested in image-based modelling.

Standard histological techniques such as tinctorial staining, Immunohistochemistry (IHC), or immunofluorescence enable quantitative microstructural and even biomarker specific analysis at histological resolution in 3D ^2, 3, 44, 45^. As µCT imaging is non-destructive, including tissue protein structure ^9^, these protocols can be carried out on tissue that has been previously scanned using µCT. Therefore, the image data of stained tissue can be correlated to the 3D context conveyed by the µCT conducted beforehand, which enables 3D assessment of biomarker localisation through correlative imaging. If serial histological data is collected, 3D features identified by biomarker expression can also be segmented, and then assessed quantitively in a 3D context.

In addressing the challenges of registration between histology and µCT, we have encountered the issue of elastic deformation during the sectioning process and tissue slice "floating." To ensure precise alignment, we have developed an elastic registration approach, which is thoroughly described in 3 and 23. This is a versatile technique, which can work between a variety of datasets, including XRH, conventional histology, immunohistochemistry, electron microscopy or other imaging modalities.

In addition, the popularity of using µCT data sets as imaging input for image-based modelling is increasing, primarily due to the increased availability of high computational power. Input geometries for modelling can be obtained by segmenting µCT data or through correlative imaging, as discussed in the previous paragraph. As a result, XRH data can be utilized to model physiological and mechanical processes, allowing for the assessment of the impact of pathological tissue remodelling.

Example research work using these bespoke visualisation methods are described in Case study 2 and Case study 3.

***Case study 2: Correlative 2D/3D visualisation and augmented tomography*.** XRH can be paired with conventional 2D light microscopy-based histology and IHC. This is further supported by preliminary data demonstrating the safety of XRH against radiation damage ^9^, as it does not seem to affect protein antigenicity. As such, XRH can be used for multimodal correlative imaging ^2, 7, 8, 30^, which offers a unique perspective into structure-function relationships of the studied tissues.

**Figure 10.**
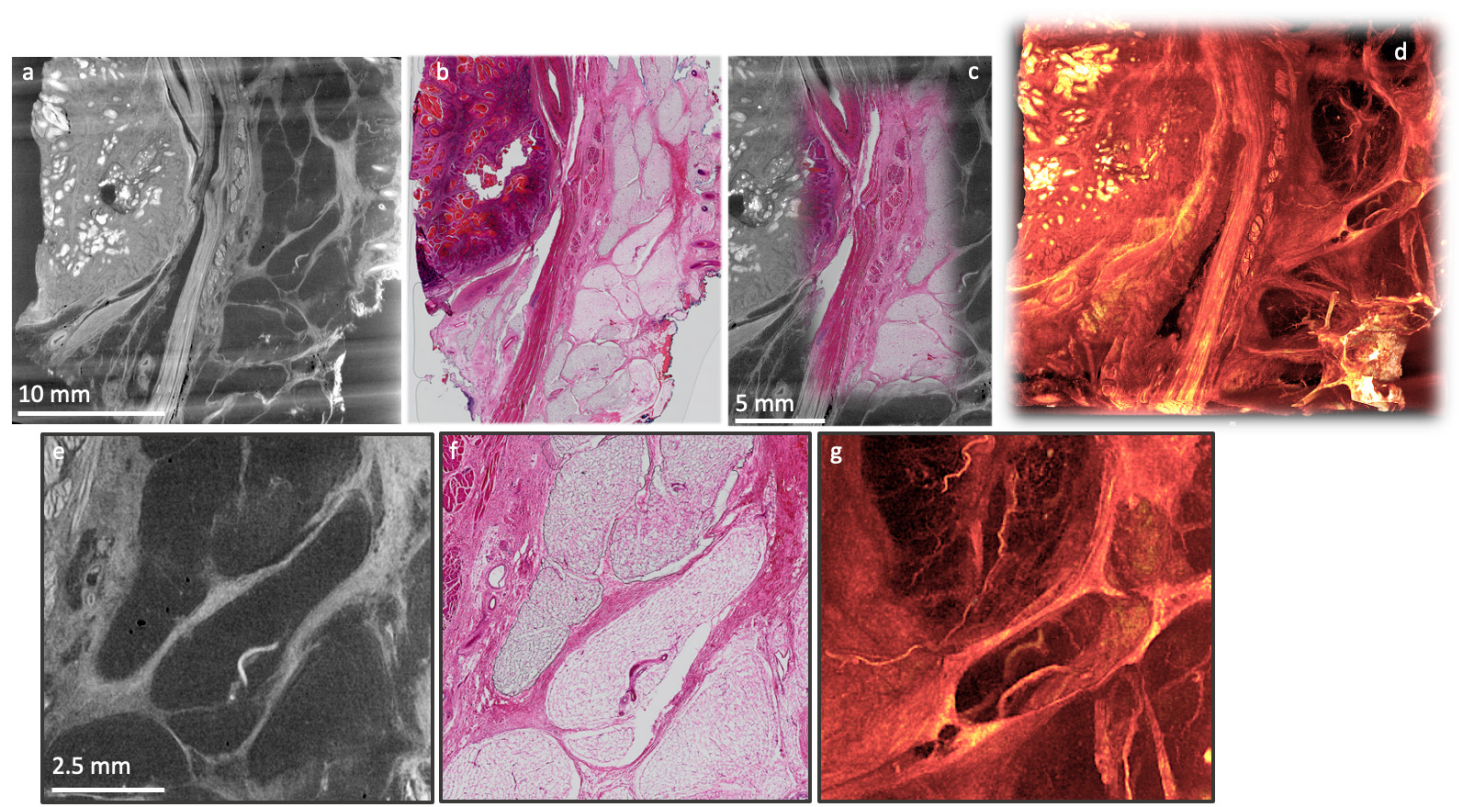
Correlative XRH and conventional histology imaging of a human head and neck tumour specimen. Specimen was scanned on a cassette at 15 μm voxel (3D pixel) size without staining. Serial sections were taken after μCT and stained with H&E. Top row: Side-by-side visualisation of matching XRH and conventional histology images on the left ( **a**, **b**); Augmented tomography in the middle ( **c**) is made possible by fusing conventional histology images with XRH data to enhance the specific information presented by the mainly structural μCT data; Volumetric visualisation of the whole tissue block on the right ( **d**) offers 3D context and overview of the microanatomy. Bottom row: Close-up images on the central part of the volume shown in ( **a**) & ( **b**). Single XRH slice on the left ( **e**), H&E slice in the middle ( **f**), and an MIP image ( **g**) uncovering the volumetric development of micro-vasculature on the right.

An example is presented here, where a human head and neck tumour specimen was imaged using both XRH and conventional histology, and the data were combined to allow for a correlative multimodal assessment of the excised specimen ( Figure 10). XRH imaging was utilised to provide a 3D overview of the entire specimen, while conventional histology allowed higher resolution 2D imaging of tissue’s microanatomy. The μCT and histology images shown in Figure 10 were matched to achieve accurate correspondence between the two modalities. The process involved rigid reslicing of the XRH data (rotate & translate) to accurately match the histology cutting plane and semi-automatic landmark-based registration of the 2D histology image onto the relevant XRH μCT slice as explained in 6. This way, powerful visualisations can be generated by overlaying conventional histology images on top of the wider field of view from the µCT scan (augmented tomography). This allows the higher resolution histology image to be viewed in the context of the wider tissue and structures. 

**Figure 11.**
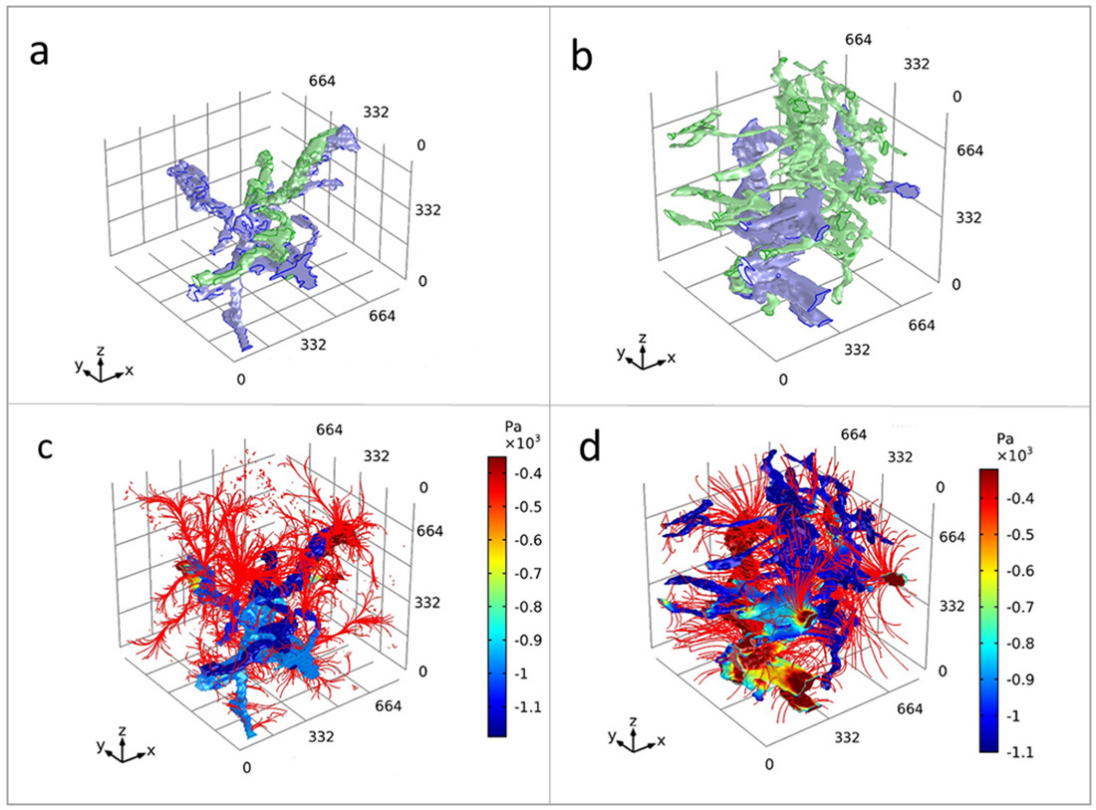
3D XRH-based mathematical modelling. 3D model simulation result for flow in an intralobular and subpleural lung geometry. Graphical representations of human lung tissue volumes of interest at an intralobular location and a subpleural location ( **a** and **b**, respectively). Blood vessels = blue, lymphatic vessel = green. Resultant mathematical model solution for the static pressure (Pa) within vasculature at an intralobular location and a subpleural location ( **c** and **d**, respectively). Streamlines of Darcy’s velocity field into the interstitium are shown in red. The interstitial pressure results have been removed from view so the streamlines could be visualised. The volume of interests has a dimension of 830 × 830 × 830 μm ^3^. A visual outline of the segmentation process of the relevant features used for generating the model shown here are illustrated in figure 2 in 30, where the segmented features (lymphatic- and blood- vessels) are rendered against a histological slice and 2D and 3D μCT images.

***Case study 3: 3D XRH-based mathematical modelling*.** µCT-based XRH has shown to be an essential tool in workflows for 3D image-based modelling ^30^. In this case study, we outline image processing methods to extract vascular networks within human lung tissue using correlative µCT and IHC methods. This provided 3D input geometries for successful computational fluid flow simulations between the blood and lymphatic vessel networks through parenchymal tissue. Due to altered 3D tissue microstructure, preliminary modelling results showed marked differences in lung drainage patterns at different anatomical locations within healthy lung tissue ( Figure 11). This work demonstrates that high-fidelity 3D isotropic geometries produced with µCT are crucial for accurate soft-tissue image-based modelling.

### IT infrastructure

A high-throughput facility, such as the 3D XRH facility, requires a high-performance IT infrastructure able to support both the data volumes and data rates produced by the µCT scanners and provide management and administration features to enable efficient operation. To manage and process large datasets, it is important to carefully consider how to handle the technical complexities. The XRH facility has made their high-performance IT infrastructure more accessible to non-experts by shielding them from some of these complexities. By adopting this approach, users are able to concentrate on the substantive aspects of their data analysis, rather than being impeded by the logistical challenges involved in data generation and management.

The new XRH XT H 225 ST scanner requires a 10 GBit/s network for acquisition of scans at the highest framerates, which is a step up compared with the previous Med-X prototype that uses a 1 GBit/s network connection. This requirement for higher network performance is due to the higher sensitivity and higher resolution detector, which can produce scans of 50 – 100 GB of radiographs. This increase in network performance also had to be matched with suitable high-speed, high-capacity storage. This storage, consisting of both SSD and HDD drives, has been deployed locally to the scanner to maximise performance and reliability. The underlying hardware only forms part of the required infrastructure. To ensure that hardware problems are detected as soon as possible, a network-monitoring system is being run to provide automatic alerts of any failures or degradation in the hardware, enabling immediate action to be taken to remedy the issue. Multiple workstations are needed to ensure that data can be processed while another scan is ongoing. Data processing at the 3D XRH facility can be carried out using a combination of open-source and commercial software as described in Section 5.4. This provision is supported by graphics tablets and virtual reality headsets. Some of the samples scanned may be further (destructively) processed after scanning, which renders it impossible to reproduce scans at a later date. This means that, apart from primary storage for the scan data, systems for backing up and recovering the data in case of drive failure also needed to be put in place.

**Figure 12.**
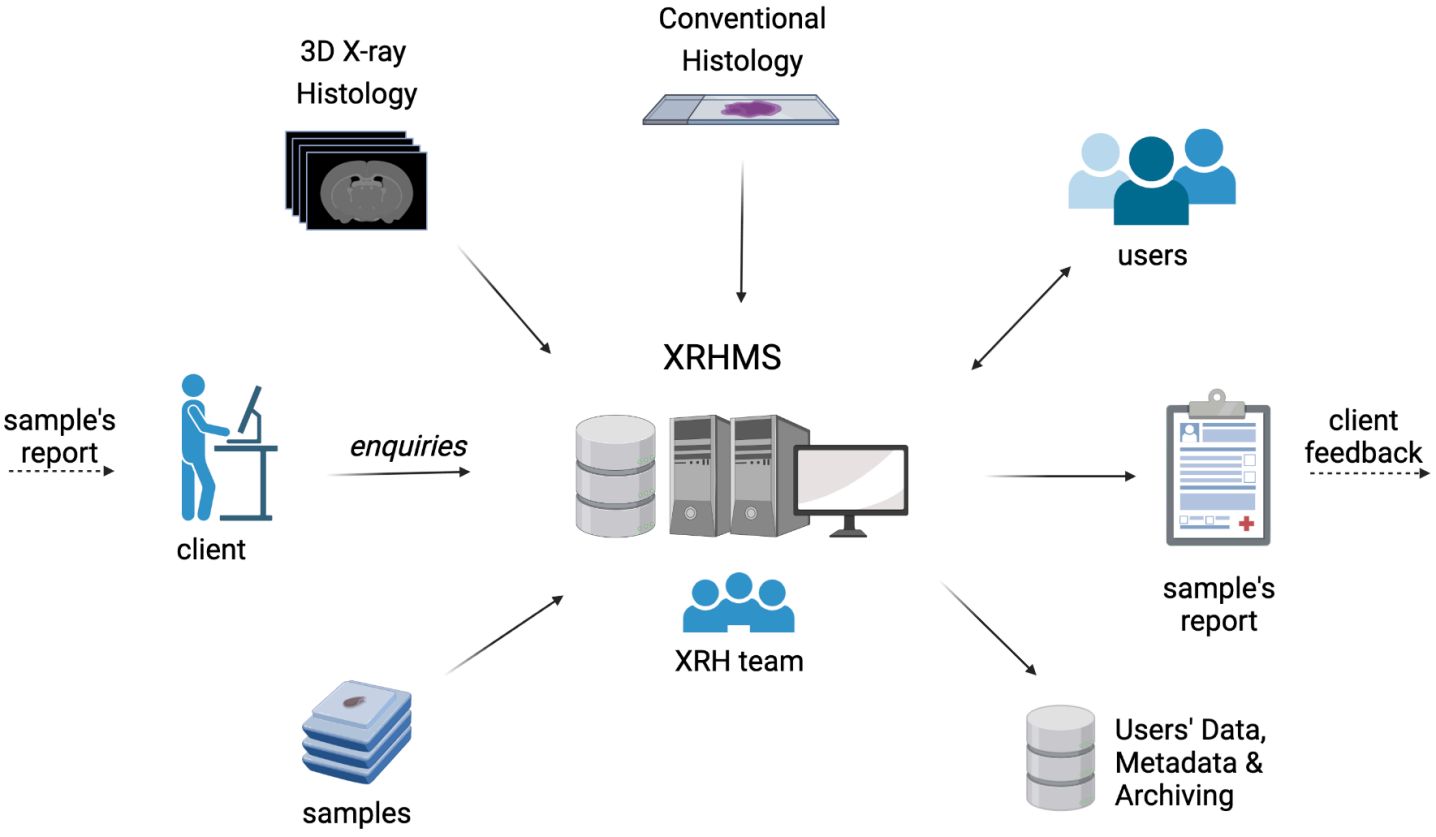
The X-ray Histology Management System (XRHMS). The XRHMS is designed to be the control centre of all operations at the 3D XRH facility. Typical inputs include new project entries, client/user enquiries as well as sample logging and tracking. Importantly, XRHMS automatically handles incoming imaging data, generates metadata, organises, and assigns these data to samples and projects.

***X-ray Histology Management System (XRHMS)*.** The μ-VIS X-ray Imaging Centre at the University of Southampton uses a custom management platform built to meet the needs of the facility ^46^. When scanning high volumes of samples, it is important to record scan parameters and metadata to ensure scan reproducibility. This management platform allows scans to be tracked and metadata to be catalogued. This system along with ideas and concepts from 47, 48 have been used to develop a new management system specifically designed for use within the XRH facility. Existing lab information management systems (LIMS) such as MetaLIMS ^49^, Baobab ^50^, and OpenLIMS ^51^ were investigated but these were either too specific to their intended application domain, not maintained, or too heavyweight incorporating various unnecessary features. The bespoke X-ray Histology Management System (XRHMS) we developed is used to track projects, samples, and generated XRH data throughout their lifetime, where a summary of this is shown in Figure 12. A key feature of the XRHMS is that it tracks a sample through multiple states, for example from being scanned as a fresh sample, then being FPPE-fixed and scanned again, before being sectioned, processed, stained, and imaged with conventional section-based histology. By tracking the sample through all states of processing, the data from each process can be aggregated to implement correlative imaging experiments, thus maximising the information available from a valuable sample. The XRHMS has been designed to also track the movement of data from online hot, and warm storage to cold archival storage. Additional options to present archived data publicly such as X-NAT ^52^ are being investigated ^53^, with other options on the roadmap for future work. Finally, the XRHMS system links to the existing μ-VIS infrastructure, enabling statistics to be generated for the facility through the μ-VIS system and facilitating data sharing between the two platforms.

***Efficient delivery of 3D datasets*.** The large file sizes of the generated datasets pose challenges not just for storage and processing, but also for sharing with collaborators. One option is to provide a USB hard-drive with all the data on it, which can be done but has several limitations. Today, the bandwidth available between universities is such that transferring a dataset online is not unreasonable, however, appropriate tools are required. One such tool is the open-source zend.to which enables arbitrarily large files to be sent to multiple recipients, within reasonable time-frames. Even when the data has been transferred to the client who requested the scan, this is not its final destination, as it should also be made available to readers if the scan results in a publication. This can be achieved by depositing the data into institutional repositories but options for online visualisation are also being explored. Another disadvantage of exporting the data to an external hard-drive is that it forms data silos, therefore scans previously taken cannot be incorporated into new projects for further analysis. This ongoing storage of previous scans poses both technological and management/authorisation challenges.

One of the challenges is finding the best way to share the 3D data generated with remote collaborators, some of which might be unfamiliar with the technology and its outputs. This created the need for easily accessible and comprehensive reporting of the XRH data, and to address this we developed a semi-automated reporting system that is linked to XRHMS. The system generates augmented Portable Document Format (PDF) reports that contain sample information, imaging settings, still images with descriptive figure legends, and links to corresponding online videos. These videos provide 3D viewing modes of the scanned samples and are easily shareable through email or other means (see supplementary materials). These videos can move through consecutive single slices of the image stack in each of the three planes XY, XZ, YX; through thick slices of processed data (MIP, Avg, StdDev); through 3D renders; or through the raw radiographs (see supplementary videos). The exact combination and parameters for video generation are currently being evaluated. Video generation is semi-automated to ensure reproducibility of results on a project-by-project basis. If a standardised set of parameters and videos is defined, this process can be fully automated (with the option of including additional videos semi-automatically).

The report also includes links to useful resources and recommendations or commentary from the XRH team. The fully formatted report can be generated with a minimum of user input before being forwarded to collaborators. The processed datasets can also be shared with collaborators online, and support for viewing and extracting all important information from them is offered.

### Client access to the 3D XRH facility and XRH project enquiry workflow

There are several pathways to access the 3D XRH facility and the services offered. One of them is through the National Research Facility for laboratory-based X ray CT (NXCT) Facility, which is the UK’s National Research Facility for lab-based X-ray CT of which the University of Southampton is one of its founding members ( nxct.ac.uk), or directly through an enquiry to the μ-VIS X-ray Imaging Centre ( www.muvis.org). The NXCT is currently offering a limited number of free at the point of access scan days to academic, non-profit and SME clients to carry out proof of principle studies. Larger or more complicated projects that go beyond a proof-of-principle basis need to be costed, and there are procedures in place for offering such access to both academic and industrial partners. For academic clients of the facility the cost is calculated using a Full Economic Costing (FEC) model, for industrial partners it is costed on a commercial basis. In either case the clients have exactly the same access to the facilities and the staff within, the client is welcome to come and see the facility and when developing a protocol it can be helpful to have them on site to provide real-time feedback, equally we have clients where the entire process has been carried out with them in a different country.

**Figure 13.**
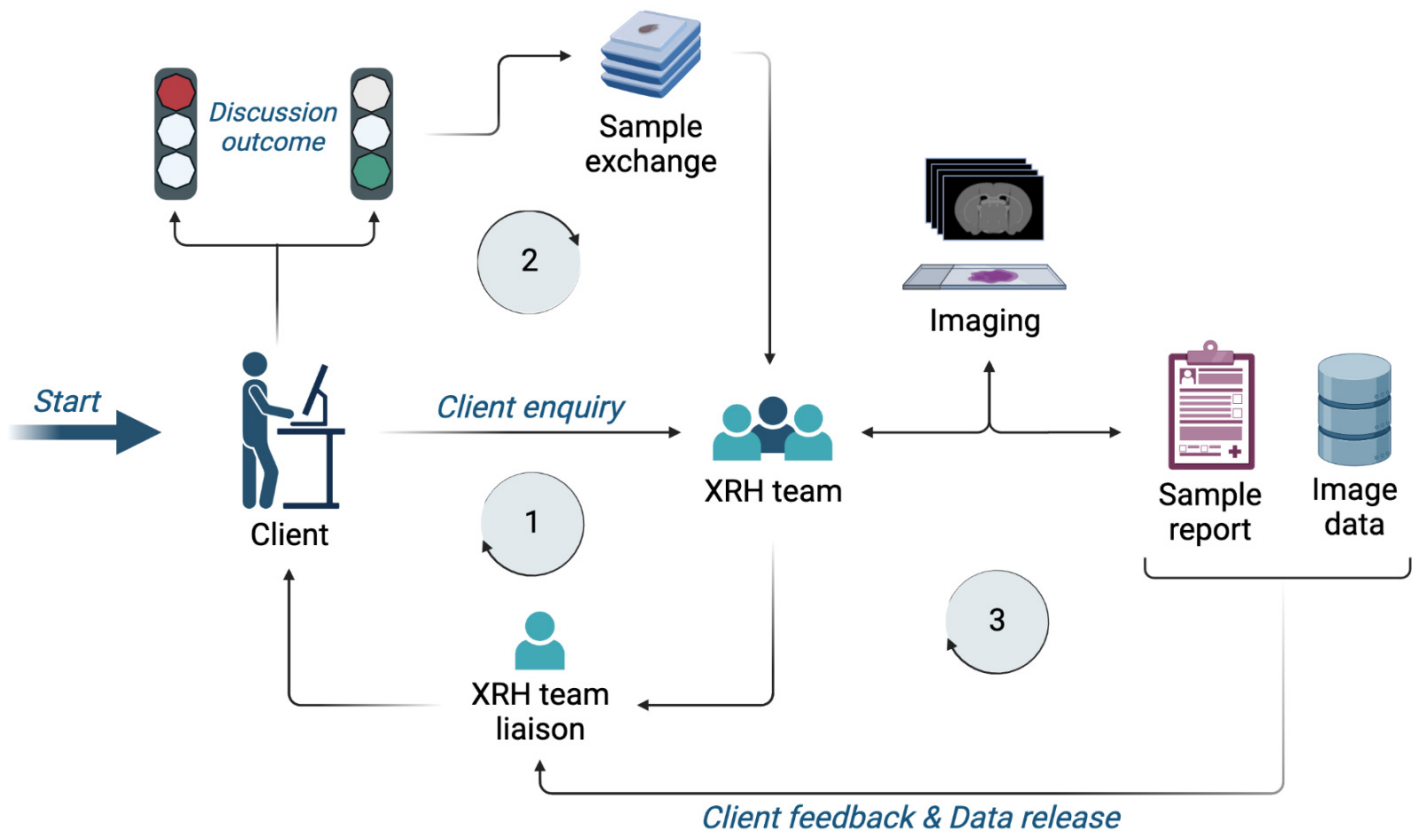
XRH facility access request workflow. **Stage 1**: An initial enquiry is submitted to the XRH team, which is getting in contact with the client to discuss project details and assess feasibility. **Stage 2**: If feasibility criteria are met, samples are sent to the facility and project commences. **Stage 3**: Imaging, reporting, and data release to the client. Depending on the project design, this stage may also include complementary sample processing, conventional histology, correlative imaging and/or bespoke data analysis.

An XRH enquiry from a client ( Figure 13) through any accessing pathways will be followed up by the XRH team, who will discuss requirements, feasibility, and deliverables with the client. Once project objectives are agreed, samples will be sent to the facility for imaging, following setup of appropriate documentation. The samples would normally arrive in the facility processed, embedded in paraffin wax, and mounted on a histology cassette, although other sample states such as frozen, wet-fixed or fresh are also acceptable. For most applications, FFPE samples are imaged on the cassette to minimise sample manipulation. The standard range of equipment allows imaging at spatial resolution down to 5 μm, although higher resolutions can be achieved with minor modifications of the hardware. However, higher resolution sacrifices a larger field of view, which enables whole-block imaging and 3D visualisation of the entire tissue. XRH has been designed to seamlessly fit into established histology workflows. Consequently, complementary conventional histology and/or IHC can also be performed following imaging at the 3D XRH facility, as in the case of correlative imaging projects, either in specialised labs of the user or on site at the Histochemistry Research Unit ( www.southampton.ac.uk/hru) at the University of Southampton. All samples, data and metadata are being tracked and interlinked using the XRHMS described earlier and in 54, which is designed to orchestrate the whole entire workflow. XRHMS empowers the facility scientists to fully track the journey of all samples, their associated data and metadata and interlink these with the respective projects and people involved. This system is vital for generating the sample scan reports ^55^ and collating client data for release before archiving. This combination of 3D X-ray histology, histochemistry and optical and electro-magnetic imaging research facility is unavailable anywhere else, and provides a unique and powerful combination that can be used to further clients research.

## Discussion & Conclusions

Our 3D X-ray Histology facility can assist biomedical research and clinical practice across many disciplines. 3D XRH offers amongst others (1) micrometre resolution in 3D, which allows visualisation and quantification of tissues’ microstructure in their 3D context, (2) a non-destructive approach to tissue histology that makes it suitable for correlative imaging, e.g. with specific 2D histology techniques such as histochemistry and immunocytochemistry, and (3) high image contrast for non-stained FFPE specimens.

The XRH facility is currently capable of scanning FFPE, fresh, and wet tissue, and to also do quick scans of frozen samples. These quick-frozen scans can be performed by using dry ice to provide a curtain of cold air that sinks over the sample to keep it cool. This approach is simple, inexpensive and produces good results, but it has limitations if a long scanning time is required. Dry ice can cause a condensation build up around the sample which can affect image quality, and it also ‘runs out’ (sublimates) after a relative short period of time; i.e. 15 –30 mins depending on the size of the reservoir. To resolve this issue and extend our ‘cryo’-imaging capabilities a liquid nitrogen -based system has just been purchased to allow for longer scans (> 5h) of frozen samples. This system will use a jacket of dry air around the sample which is anticipated to reduce any issues due to condensation.

Although our team’s primary focus in Southampton has been on biomedical material such as clinical and animal tissues, the 3D XRH facility has been also used for samples from other sources ranging from sea corals to biofilms, and from meat substitutes for food technology advancements to pharmaceutical technology related to novel dosage forms. In the past 7 years, we have shown that it is possible to set up a facility for the μCT imaging community, with successful completion and initiation of over 120 projects involving 125+ collaborators from 60 affiliations. This has resulted in 15 published manuscripts, 10 submitted/in preparation, over 35 conference proceedings, and over 25 research funding proposals ^1– 3, 7, 23, 30– 32, 37, 44, 56– 59^.

The technological developments produced while setting up the 3D XRH facility improve standard µCT imaging methods by eliminating engineering complexities. Systems have been developed to support and automate processing, thus, improving productivity. Users can acquire 3D datasets quickly and easily with minimal involvement if they so wish. However, it is important to highlight that as with any imaging technique there is never a perfect imaging protocol for every application. Users’ expectations must be carefully managed before undertaking scanning work. The factors that must be balanced include spatial and contrast resolution, image noise and artefact control against throughput speed. The throughput speed has the largest impact on cost from both the facility and users’ point of view and can be a make-or-break point for many applications. Furthermore, in the context of µCT imaging and its correlative applications, ensuring specimen quality is of utmost importance. For example, a notable challenge in FFPE specimens of soft tissues is the presence of air bubbles resulting from suboptimal wax embedding. Strategies to address this issue include customised post-processing algorithms that effectively mitigate interference artifacts arising from the bubble-wax interfaces, or when necessary dewaxing and re-embedding. These steps ensures that, in most of the cases, the processed data is suitable for automatic thresholding, enhancing the accuracy of subsequent analyses.

Another point of consideration that should be addressed when discussing experimental planning with the client is the prospect of radiation-induced specimen damage. Such damage has the potential to influence downstream biochemical analyses, including immunohistochemistry, proteomics, and transcriptomics. Notably, existing research in this area has predominantly focused on synchrotron radiation-based studies, where substantial doses have been found to impact tertiary protein structures ^60^. However, for laboratory-based radiation doses, especially within the context of the low-dose protocols typically used for soft tissue imaging in our facility (0.1 –5.0 kGy), there appears to be minimal impact on the tissue, especially concerning the affinity of tissues for histological stains ^9^. Nevertheless, in the near future, we will focus on assessing protein and RNA quality to ensure the preservation of molecular integrity. This step is crucial in achieving a truly "non-destructive" technique at all hierarchical levels, which is essential for facilitating correlative biochemical analyses.

At this point, it is important to note that, while the non-destructive/non-intrusive nature of XRH imaging holds true for the X-ray imaging component of the workflow, non-destructiveness and non-intrusiveness, in the grand scheme of things, should be interpreted within the context of classical histology workflows and after the FFPE stage. Preceding stages, including sample preparation and paraffin embedding, are elements that fall outside the direct control of the workflow discussed here, as our approach is designed to primarily handle specimens that have undergone the classical sample preparation journey. However, we should highlight that classical tissue handling, including the processes leading to FFPE, can affect tissues and introduce disturbances such as tissue shrinkage. More specifically, the FFPE process induces tissue dehydration, resulting in both global and local tissue shrinkage and densification. The dehydration steps in FFPE play a pivotal role in increasing the molecular density per unit volume. This is primarily attributed to the removal of substantial quantities of hydrogen and oxygen elements from the sample, leading to a densification of the organic elements proportionate to their hydration level. This densification contributes to subtle distinctions between elements with varying degrees of hydration ^61, 62^.

Understanding the physical effect FFPE processing has on tissues can offer insights into the quality and interpretability of X-ray histology images. In the realm of soft-tissue imaging, particularly within the context of FFPE tissue, the majority of components consist of organic material with comparable attenuation coefficients. Interestingly, non-staining XRH protocols exploits minute X-ray attenuation variations introduced by the very process of FFPE processing. Alongside the discussed densification, formaldehyde-based fixatives also induce cross-linking of native tertiary protein structures ^61, 63^ linking a great number of molecular subunits including those that are relatively far apart ^64^. We believe that these cross-linking bonds increase localised electron density, which in turn, affects their X-ray attenuation properties. Regions with heightened cross-linking appear "denser" than their non- or less-cross-linked counterparts. Similarly, the variable densification between tissue components caused by dehydration also contributes to X-ray contrast resolution. These subtle attenuation differences are what attenuation-based XRH of FFPE specimens exploits for contrast. However, further work is needed to systematically collect data and identify the contribution of each processing stage to enhanced sample contrast and investigate the impact of common fixatives on contrast levels in histological protocols.

The impact of formalin fixation on tissue dimensions is a critical consideration in various studies, as such, it should be noted for those wanting to use our facility for absolute native tissue architecture. Formalin fixation is known to induce shrinkage in cancerous tissue, potentially leading to the underestimation of tumour staging and studies across different organs, such as the oesophagus, prostate, breast, lung, and cervix, have reported varying degrees of shrinkage following formalin fixation. For instance, oesophageal tumours have demonstrated a 10% shrinkage, while the overall shrinkage of the entire oesophageal specimen reached 50%. Prostate, breast and head and neck cancer specimens experienced an average linear shrinkage of up to 4.5%. The degree of shrinkage appears to vary among studies, suggesting that tissue constitution, including the presence of muscle or fat tissues, may influence the overall shrinkage ^65^. It should be said, however, that the majority of our clients to date use relative measures and their study sample groups and controls have undergone the same level of processing.

There are also major challenges around how to distribute, annotate and archive µCT datasets. The current process of either using online transfer or shipping of hard disks is suitable for point-to-point transfer of data but has major limitations. One of the issues has been the number of different file formats produced by µCT scanners, within the µ-VIS facility we have scanners from multiple manufacturers each of which uses a different output format. This has previously been dealt with by converting into either tiff stacks or raw volumes to enable data exchange. Newer file formats such as hdf5 ^66^ or OME-NGFF ^67^ as implemented by OME-ZARR ^68^, may facilitate easier transfer of the raw data and associated metadata between sites, using software such as MoBie ^69^. Other options for sharing the data online such as webknossos ^70^, which was designed for 3DEM images, or Horos cloud (Horos, https://horosproject.org/). It is envisioned that eventually that there will be tooling and practices to enable 3D XRH data to be easily shared according to the FAIR data principles ^71^. Below we outlined some of the more-prominent challenges associated with managing clients’ project at the XRH facility:

- **Imaging Trade-offs:** Balancing the trade-off between field of view and resolution, as well as resolution to scanning time (and thus cost), poses a challenge that needs to be carefully considered.
- **Data Size Challenges:** The size of data generated poses challenges for curation, archiving, and open-data strategies, particularly in studies requiring high *n* numbers. Additionally, ambiguity regarding what constitutes raw data (raw uncorrected collected radiographs, corrected radiographs, float-point reconstructions, processed 16-bit / 8-bit volumes…?) introduces an extra layer of complexity.
- **Complexity of Image Analysis:** Managing the intricacies of image analysis, particularly in terms of quantification, presents a significant challenge to many users, especially the ones with no or limited experience to processing of volume data.
- **Need for Correlative Imaging:** Correlative imaging is often required for biomarker identification, which adds an additional layer of complexity (and cost) to the workflow.
- **Contrast Challenges with Non-Stained Soft Tissues:** The majority of non-stained soft tissues, when imaged in a hydrated state exhibit minimal X-ray contrast. For such specimens other approaches are much better suited, and should be considered including phase-contrast imaging ^12, 72^, contrast CT ^19^ and SRCT ^17^.
- **Cost of Relevant Equipment:** The acquisition and maintenance costs associated with the necessary equipment capable of handling XRH data, including software and network infrastructure, can be substantial and lead to underutilisation of the data. For this reason, it is important that an XRH facility should be prepared to invest and offer access to processing infrastructure.
- **Standardisation and DICOM Compatibility:** Ensuring standardisation and compatibility with DICOM (Digital Imaging and Communications in Medicine) standards remains a crucial consideration, and one that can unlock clinical compatibility.

Despite of the aforementioned challenges, XRH is slowly being established as a clinical tool. The team at the Department of Pathology, Memorial Sloan Kettering Cancer Center in New York, USA, which had been one of the very early adopters of XRH, have already taken a significant step towards clinical integration by issuing a standard operating procedure (SOP) of μCT pathology ^9^. We envisage this to be directly translated into a next-generation clinical image-based diagnostics and a patient stratification tool.

μCT imaging has been also utilised in a clinical context for *μCT-based foetal post-mortem autopsy* ^73– 76^. In the UK, the technique has been pioneered and it is currently offered as a service at Great Ormond Street Childrens' Hospital (GOSH), London, UK by Dr Owen Arthurs et al, who have successfully conducted more than 700 investigations to date. The technique is summarised in 73 and there is also a significant volume of published articles from the team describing the development, validation, and integration workflows ^77– 79^.

In addition, by integrating XRH as a scouting tool, one can preview and identify the optimal slicing location(s) before tissue sectioning, ensuring precise and representative sampling. This approach, which we call XRH-guided histology, holds immense potential to improve the accuracy of diagnosis, enhance patient outcomes, and revolutionize the way patient samples are analysed in the clinic (see Figure 1).

The use of non-stained FFPE specimens enables histomics, i.e., the study of tissue structure-function relationships by means of advanced microscopy techniques. On this account, by exploiting archival material stored in many hospitals and tissue banks using XRH, microstructural hallmarks of disease seen in XRH data can be fully validated in the future. This will accelerate the uptake of XRH as biomedical research tool as well as a promising technology for pre-clinical and clinical studies.

In conclusion, the 3D X-ray Histology (XRH) facility at the University Hospital Southampton (UHS) holds a truly unique position in the field, distinguishing itself as the world's only dedicated XRH facility that oversees every stage of the imaging process, from user support to data generation, analysis, user training, data archiving, and metadata generation. Its integration within a clinical environment at UHS further enhances its significance, offering unparalleled opportunities for clinical applications and collaborations. Furthermore, the facility introduces a multitude of novel aspects to the realm of histology and imaging. With state-of-the-art technology, user-friendly interfaces, and a diverse range of applications, it stands at the forefront of innovation. The facility's capacity for micrometre-resolution 3D imaging enables the visualization and quantification of tissue microstructures in their full three-dimensional context, unlocking unprecedented insights, while seamlessly fits in conventional histology workflows. Its non-destructive approach to tissue histology allows for correlative imaging with specific 2D histology techniques such as histochemistry and immunocytochemistry, expanding the analytical possibilities. These aspects position the 3D XRH facility at UHS as an exceptional platform for advancing research and clinical practice in the study of tissue structure-function relationships. This article provides a comprehensive template for establishing a similar 3D X-ray Histology (XRH) facility, covering some of the key aspects. It serves as a resource for researchers and institutions interested in developing state-of-the-art facilities dedicated to advancing histology and imaging techniques. Researchers looking to take advantage of this resource or considering setting up a similar facility, are encouraged to contact and/or apply for access. With access to state-of-the-art equipment, a supportive community of experts, the new imaging facility is a valuable platform for exploring new frontiers in research and discovery.

## Ethical statement

Case study 1 and all relevant experimentation protocols were approved by and conducted in the authorized animal facilities of the National Hellenic Research Foundation, Greece. It was complied with the Protocol on the Protection and Welfare of Animals, the regulations of the National Bioethics Committee, and the Article 3 of the Presidential Decree 160/1991 (in line with 86/609/EEC directive), regarding the protection of experimental animals as per in 32. CASE STUDIES 2 and 3 were conducted with full ethical approval from the National Research Ethics Service Committee, South Central - Southampton A in the UK (numbers 09/H0501/90 and 08/H0502/32) respectively, with signed informed consent. All methods were performed in accordance with the guidelines and regulations of the National Research Ethics Service Committee as per 30.

## Data Availability

Zenodo. “A high-throughput 3D X-ray histology facility for biomedical research and preclinical applications - Supplementary Data” https://doi.org/10.5281/zenodo.10185486 ^80^ - This project contains the following supplementary data: Videos 1-7 for Version 1 of 'A high-throughput 3D X-ray histology facility for biomedical research and preclinical applications'

**Video 1** (A video going through the Z stack in single slices. This is a cross- sectional view of the XRH image stack along the XY plane. XRH datasets are normally oriented (resliced) in a way that a scroll through the stack along the XY plane emulates the physical histology slicing of the tissue).
**Video 2 (**A video going through the Y stack in single slices. This is a cross- sectional view of the XRH image stack along the XZ plane. XRH datasets are normally oriented (resliced) in a way that a scroll through the stack along the XY plane emulates the physical histology slicing of the tissue).
**Video 3 (**A video going through the X stack in single slices. This is a cross- sectional view of the XRH image stack along the YZ plane. XRH datasets are normally oriented (resliced) in a way that a scroll through the stack along the XY plane emulates the physical histology slicing of the tissue).
**Video 4 (**3D X-ray histology (XRH) is a µCT -based workflow tailored to fit seamlessly into current histology workflows in biomedical and pre-clinical research, as well as clinical histopathology. Microanatomical detail can be captured from standard (non-stained) formalin-fixed and paraffin-embedded (FFPE) tissue blocks).
**Video 5** (Average Intensity Projection (AIP) of the sample through the Histologically relevant plane. This is a 2D visualisation rendering the Average Intensity of 20x single XY slices along the z-axis of the stack. XRH datasets are normally oriented (resliced) in a way that a scroll through the stack along the XY plane emulates the physical histology slicing of the tissue).
**Video 6 (**Maximum Intensity Projection (MIP) of the sample through the Histologically relevant plane. This is a 2D visualisation rendering the Maximum Intensity of 20x single XY slices along the z-axis of the stack. XRH datasets are normally oriented (resliced) in a way that a scroll through the stack along the XY plane emulates the physical histology slicing of the tissue).
**Video 7 (**Standard deviation projection of the sample going through the histologically relevant plane. This is a 2D visualisation rendering the Standard Deviation of 20x single XY slices along the z- axis of the stack. XRH datasets are normally oriented (resliced) in a way that a scroll through the stack along the XY plane emulates the physical histology slicing of the tissue).

*** ***Videos 5 –7*** *(Also referred to as "thick-slice rolls"* - *Thick-slice rolling is a 2D thick-slice viewing that allows rolling of a pre-selected number of slices (n) along the z-axis of the 3D data. A single thick-slice roll forwards is accomplished by translating the thick-slice by one single slice forwards; that is moving forward by one (+1) slice from the first and nth element and reapplying the criteria or operations to the new slice sub-stack).*
Click here for additional data file.Copyright: © 2023 Katsamenis OL et al.2023 **Video 1** (A video going through the Z stack in single slices. This is a cross- sectional view of the XRH image stack along the XY plane. XRH datasets are normally oriented (resliced) in a way that a scroll through the stack along the XY plane emulates the physical histology slicing of the tissue).
**Video 2 (**A video going through the Y stack in single slices. This is a cross- sectional view of the XRH image stack along the XZ plane. XRH datasets are normally oriented (resliced) in a way that a scroll through the stack along the XY plane emulates the physical histology slicing of the tissue).
**Video 3 (**A video going through the X stack in single slices. This is a cross- sectional view of the XRH image stack along the YZ plane. XRH datasets are normally oriented (resliced) in a way that a scroll through the stack along the XY plane emulates the physical histology slicing of the tissue).
**Video 4 (**3D X-ray histology (XRH) is a µCT -based workflow tailored to fit seamlessly into current histology workflows in biomedical and pre-clinical research, as well as clinical histopathology. Microanatomical detail can be captured from standard (non-stained) formalin-fixed and paraffin-embedded (FFPE) tissue blocks).
**Video 5** (Average Intensity Projection (AIP) of the sample through the Histologically relevant plane. This is a 2D visualisation rendering the Average Intensity of 20x single XY slices along the z-axis of the stack. XRH datasets are normally oriented (resliced) in a way that a scroll through the stack along the XY plane emulates the physical histology slicing of the tissue).
**Video 6 (**Maximum Intensity Projection (MIP) of the sample through the Histologically relevant plane. This is a 2D visualisation rendering the Maximum Intensity of 20x single XY slices along the z-axis of the stack. XRH datasets are normally oriented (resliced) in a way that a scroll through the stack along the XY plane emulates the physical histology slicing of the tissue).
**Video 7 (**Standard deviation projection of the sample going through the histologically relevant plane. This is a 2D visualisation rendering the Standard Deviation of 20x single XY slices along the z- axis of the stack. XRH datasets are normally oriented (resliced) in a way that a scroll through the stack along the XY plane emulates the physical histology slicing of the tissue). **Video 1** (A video going through the Z stack in single slices. This is a cross- sectional view of the XRH image stack along the XY plane. XRH datasets are normally oriented (resliced) in a way that a scroll through the stack along the XY plane emulates the physical histology slicing of the tissue). **Video 2 (**A video going through the Y stack in single slices. This is a cross- sectional view of the XRH image stack along the XZ plane. XRH datasets are normally oriented (resliced) in a way that a scroll through the stack along the XY plane emulates the physical histology slicing of the tissue). **Video 3 (**A video going through the X stack in single slices. This is a cross- sectional view of the XRH image stack along the YZ plane. XRH datasets are normally oriented (resliced) in a way that a scroll through the stack along the XY plane emulates the physical histology slicing of the tissue). **Video 4 (**3D X-ray histology (XRH) is a µCT -based workflow tailored to fit seamlessly into current histology workflows in biomedical and pre-clinical research, as well as clinical histopathology. Microanatomical detail can be captured from standard (non-stained) formalin-fixed and paraffin-embedded (FFPE) tissue blocks). **Video 5** (Average Intensity Projection (AIP) of the sample through the Histologically relevant plane. This is a 2D visualisation rendering the Average Intensity of 20x single XY slices along the z-axis of the stack. XRH datasets are normally oriented (resliced) in a way that a scroll through the stack along the XY plane emulates the physical histology slicing of the tissue). **Video 6 (**Maximum Intensity Projection (MIP) of the sample through the Histologically relevant plane. This is a 2D visualisation rendering the Maximum Intensity of 20x single XY slices along the z-axis of the stack. XRH datasets are normally oriented (resliced) in a way that a scroll through the stack along the XY plane emulates the physical histology slicing of the tissue). **Video 7 (**Standard deviation projection of the sample going through the histologically relevant plane. This is a 2D visualisation rendering the Standard Deviation of 20x single XY slices along the z- axis of the stack. XRH datasets are normally oriented (resliced) in a way that a scroll through the stack along the XY plane emulates the physical histology slicing of the tissue). *** ***Videos 5 –7*** *(Also referred to as "thick-slice rolls"* - *Thick-slice rolling is a 2D thick-slice viewing that allows rolling of a pre-selected number of slices (n) along the z-axis of the 3D data. A single thick-slice roll forwards is accomplished by translating the thick-slice by one single slice forwards; that is moving forward by one (+1) slice from the first and nth element and reapplying the criteria or operations to the new slice sub-stack).* Click here for additional data file. **Videos:** **Video 1** (A video going through the Z stack in single slices. This is a cross- sectional view of the XRH image stack along the XY plane. XRH datasets are normally oriented (resliced) in a way that a scroll through the stack along the XY plane emulates the physical histology slicing of the tissue). **Video 2** (A video going through the Y stack in single slices. This is a cross- sectional view of the XRH image stack along the XZ plane. XRH datasets are normally oriented (resliced) in a way that a scroll through the stack along the XY plane emulates the physical histology slicing of the tissue). **Video 3** (A video going through the X stack in single slices. This is a cross- sectional view of the XRH image stack along the YZ plane. XRH datasets are normally oriented (resliced) in a way that a scroll through the stack along the XY plane emulates the physical histology slicing of the tissue). **Video 4** (3D X-ray histology (XRH) is a µCT -based workflow tailored to fit seamlessly into current histology workflows in biomedical and pre-clinical research, as well as clinical histopathology. Microanatomical detail can be captured from standard (non-stained) formalin-fixed and paraffin-embedded (FFPE) tissue blocks). **Video 5** (Average Intensity Projection (AIP) of the sample through the Histologically relevant plane. This is a 2D visualisation rendering the Average Intensity of 20x single XY slices along the z-axis of the stack. XRH datasets are normally oriented (resliced) in a way that a scroll through the stack along the XY plane emulates the physical histology slicing of the tissue). **Video 6** (Maximum Intensity Projection (MIP) of the sample through the Histologically relevant plane. This is a 2D visualisation rendering the Maximum Intensity of 20x single XY slices along the z-axis of the stack. XRH datasets are normally oriented (resliced) in a way that a scroll through the stack along the XY plane emulates the physical histology slicing of the tissue). **Video 7** (Standard deviation projection of the sample going through the histologically relevant plane. This is a 2D visualisation rendering the Standard Deviation of 20x single XY slices along the z- axis of the stack. XRH datasets are normally oriented (resliced) in a way that a scroll through the stack along the XY plane emulates the physical histology slicing of the tissue). ** **Videos 5 –7** (Also referred to as "thick-slice rolls"* - *Thick-slice rolling is a 2D thick-slice viewing that allows rolling of a pre-selected number of slices (n) along the z-axis of the 3D data. A single thick-slice roll forwards is accomplished by translating the thick-slice by one single slice forwards; that is moving forward by one (+1) slice from the first and nth element and reapplying the criteria or operations to the new slice sub-stack).* **The questionnaire used to collect feedback about the needs of the XRH community:** Survey.docx Survey.pdf **Exemplar report of a semi-automatically generated augmented PDF file** that contain sample information, imaging settings, still images with descriptive figure legends, and links to corresponding online videos: DEMO02019-FFPE_report_99EbPXG.pdf **System performance data ZIP** that contain imaging data collected through different systems and setups at the XRH facility at the μ-VIS X-ray Imaging Centre at the University of Southampton for the purpose of acceptance and/or system performance characterisation. Below is an overview of the folder structure and its contents. The following files are X-ray imaging data collected on September 28, 2017, using the Med-X system and a Jima phantom at 55 kV peak and 7 Watts. 20170928_MEDX_1642_JIMA_55kVp7W-2.tif 20170928_MEDX_1642_JIMA_55kVp7W.tif 20170928_MEDX_1642_JIMA_55kVp7W.tif.profile.xml **A QRM MicroCT bar pattern phantom PDF** document and its specifications: QRM-MicroCT-Barpattern-Phantom.pdf **Graphs PNG file** showing the calculated focal-spot size as a function of the X-ray power (W) for the Molybdenum rotating target calculated using Edge Modulation function testing. The performance is then compared with the performance of the Reflection target across the same range of powers. Raw data can be found in XRH_QRM_Refl-vs-Rot-TargetComparison_SingleReconSlices_5umPixelSize folder. Test performed in July 2021: XRH_202107_MoRot-testing_EdgeModFunction-QRMrecons+RotReflCompar.png ***/ XRH-XT-H-225-ST_FocalSpots*** - this directory contains radiographic data collected using the XRH system with a JIMA phantom and MoRt (Molybdenum rotating), TT (Transmission), and Reflection targets: 20200113_XRH_Jima test MoRT 55kV 15W.tif, 20200113_XRH_Jima test MoRT 55kV 30W.tif, etc. - these files represent radiographs taken on January 13, 2020, using the XRH system, Jima phantom, MoRT target at 55 kVp and varying wattages. 20200207_XRH_JIMA 80kV TT1a.tif, 20200207_XRH_JIMA 80kV TT1b.tif, etc. - similar to the above, these files are from February 7, 2020, and use 80 kVp with a TT target. 20231115_XRH_reflW_80kVp6W.tif, 20231115_XRH_reflW_80kVp6W_02.tif, etc. - these files are from November 15, 2023, and collected using the XRH system with a Reflection target at 80 kVp and 6 Watts. ***/ XRH_QRM_Refl-vs-Rot-TargetComparison_SingleRadioFromCTs_5umPixelSize*** - this directory contains single radiographs taken with a pixel size of 5 micrometers using the Molybdenum rotating (MoRt), and the Reflection target using tungsten (W) and Molybdenum (Mo) metals. ***/ XRH_QRM_Refl-vs-Rot-TargetComparison_SingleReconSlices_5umPixelSize*** - this directory contains single reconstruction slices of the setups mentioned above. Slices are exported from CT volumes and were used for the Edge Modulation function study. For interpretation of the filenames in the folders listed above please see below and refer to specific files and folders for detailed information and results related to each imaging session: *<xx>kVp or <xx>kV* :Imaging at a peak voltage of <xx> kVp. *<y>W*        :Imaging at <y> Watts; "." is represented with "-"; i.e. 20210705_XRH_2766_PJB_TEST03552; EQPMT_W_6-9W is acquired using a power of 6.9 W *MoRt, TT, Refl*        :Molybdenum, Transmission, and Reflection targets, respectively. *_W_ and _Mo_*        :Tungsten and Molybdenum target materials. *_horiz*        :Reconstruction slices in line with the X-ray beam's propagation direction. *_vert*        :Reconstruction slices normal to the X-ray beam's propagation direction and parallel to the detector plane. Data are available under the terms of the Creative Commons Attribution 4.0 International license (CC-BY 4.0). Zenodo. “A high-throughput 3D X-ray histology facility for biomedical research and preclinical applications - Underlying Data” https://doi.org/10.5281/zenodo.8083459 - This project contains the following underlying data: **Video files and logs** Single-slice and thick-slice roll* source videos are included. Each video is accompanied by a .txt log that contains information about the source file, slice thickness, and a brief description of the visualization mode. List of files: 20211019-23h59m_20xAvgInt.mp4 20211019-23h59m_20xAvgInt.txt 20211019-23h59m_20xMaxInt.mp4 20211019-23h59m_20xMaxInt.txt 20211019-23h59m_20xStDev.mp4 20211019-23h59m_20xStDev.txt 20211019-23h59m_XYSliceRoll.mp4 20211019-23h59m_XYSliceRoll.txt 20211019-23h59m_XZSliceRoll.mp4 20211019-23h59m_XZSliceRoll.txt 20211019-23h59m_YZSliceRoll.mp4 20211019-23h59m_YZSliceRoll.txt * *Thick-slice rolling is a 2D thick-slice viewing that allows rolling of a pre-selected number of slices (n) along the z-axis of the 3D data. A single thick-slice roll forwards is accomplished by translating the thick-slice by one single slice forwards; that is moving forward by one (+1) slice from the first and nth element and reapplying the criteria or operations to the new slice sub-stack.* **Volume XRH data** These are processed raw volume file saved in .raw and/or .tiff format, which are resliced to a histology-relevant orientation and/or have been enhanced using noise reduction (3D median filter) and/or ct-artefact removal techniques (e.g. cBC identifies a bandpass filter used to remove intensity variations originating from the histology cassette). List of volume files: **32220_20200703_XRH_2504_OLK_DEMO02019-FFPE_1620x1959x164x16bit.raw** sample: Human lung adenocarcinoma histology-relevant resliced volume (2x2x2 3D medial filter applied) import as 1620 x 1959 x 164 x 16-bit, big-endian; voxel edge size (mm): 0.0160042 isotropic **cBC_32220_20200703_XRH_2504_OLK_DEMO02019-FFPE_1588x1674x164x16bit.raw** sample: Human lung adenocarcinoma cassette artefacts background correction (bandpass) of volume 32220_20200703_XRH_2504_OLK_DEMO02019-FFPE_1620x1959x164x16bit.raw import as 1620 x 1959 x 164 x 16-bit, big-endian; voxel edge size (mm): 0.0160042 isotropic **Med3D_HPass_2111_20190606_MEDX_2234_EH_HN2_recon_2000x1952x501x32bit.raw** sample: Human head and neck tumour histology-relevant resliced volume (1x1x1 3D medial filter applied) import as 2000 x 1952 x 501 x 32-bit, big-endian; voxel edge size (mm): 0.00999782 isotropic **Conventional Histology and correlative imaging** **HN2_Level001_MEDX080_Manual_BW_Series4.tif** H&E histology slice of the human head and neck tumour sample shown in "Med3D_HPass_2111_20190606_MEDX_2234_EH_HN2_recon_2000x1952x501x32bit.raw" **HN2_Level001_MEDX080_Manual_BW** manual landmark selection used for registering the conventional histology slice onto the μCT slice **HN2_MEDX_rotated_0080.tif** Slice 80 from volume "Med3D_HPass_2111_20190606_MEDX_2234_EH_HN2_recon_2000x1952x501x32bit.raw" that corresponds to histological slice "HN2_Level001_MEDX080_Manual_BW"

## Videos

***Video 1*** *A video going through the Z stack in single slices. This is a cross- sectional view of the XRH image stack along the XY plane. XRH datasets are normally oriented (resliced) in a way that a scroll through the stack along the XY plane emulates the physical histology slicing of the tissue.*
***Video 2*** *A video going through the Y stack in single slices. This is a cross- sectional view of the XRH image stack along the XZ plane. XRH datasets are normally oriented (resliced) in a way that a scroll through the stack along the XY plane emulates the physical histology slicing of the tissue.*
***Video 3*** *A video going through the X stack in single slices. This is a cross- sectional view of the XRH image stack along the YZ plane. XRH datasets are normally oriented (resliced) in a way that a scroll through the stack along the XY plane emulates the physical histology slicing of the tissue.*
***Video 4*** *3D X-ray histology (XRH) is a µCT -based workflow tailored to fit seamlessly into current histology workflows in biomedical and pre-clinical research, as well as clinical histopathology. Microanatomical detail can be captured from standard (non-stained) formalin-fixed and paraffin-embedded (FFPE) tissue blocks.*
***Video 5*** *Average Intensity Projection (AIP) of the sample through the Histologically relevant plane. This is a 2D visualisation rendering the Average Intensity of 20x single XY slices along the z-axis of the stack. XRH datasets are normally oriented (resliced) in a way that a scroll through the stack along the XY plane emulates the physical histology slicing of the tissue.*
***Video 6*** *Maximum Intensity Projection (MIP) of the sample through the Histologically relevant plane. This is a 2D visualisation rendering the Maximum Intensity of 20x single XY slices along the z-axis of the stack. XRH datasets are normally oriented (resliced) in a way that a scroll through the stack along the XY plane emulates the physical histology slicing of the tissue.*
***Video 7*** *Standard deviation projection of the sample going through the histologically relevant plane. This is a 2D visualisation rendering the Standard Deviation of 20x single XY slices along the z- axis of the stack. XRH datasets are normally oriented (resliced) in a way that a scroll through the stack along the XY plane emulates the physical histology slicing of the tissue.*

Data are available under the terms of the Creative Commons Attribution 4.0 International license (CC-BY 4.0).

## Other data

Some figures presented in this manuscript is a reproduction of previously published material. Readers requiring access to these data are advised to contact the corresponding authors of the original publications. Namely:

- Case study 1: Quantitative imaging for cancer research/diagnosis; Figure 9: Figure material reproduced with permission from C. Karavasili *et al.*, "Synergistic Antitumor Potency of a Self-Assembling Peptide Hydrogel for the Local Co-delivery of Doxorubicin and Curcumin in the Treatment of Head and Neck Cancer," (in eng), Mol Pharm, vol. 16, no. 6, pp. 2326–2341, 06 2019, doi: 10.1021/acs.molpharmaceut.8b01221.
- Case study 3: 3D XRH-based mathematical modelling; Figure 11: Figure material reproduced with permission from S. K. Robinson, J. J. Ramsden, J. Warner, P. M. Lackie, and T. Roose, "Correlative 3D Imaging and Microfluidic Modelling of Human Pulmonary Lymphatics using Immunohistochemistry and High-resolution μCT," (in En), Scientific Reports, OriginalPaper vol. 9, no. 1, pp. 1–14, 2019-04-23 2019, doi: 10.1038/s41598-019-42794-7.

### XRHMS source code

The code can be accessed at https://doi.org/10.5281/zenodo.8178308
which links to https://github.com/muvis-tomography/xrhms/tree/v1.0.0
License: Apache License 2.0

Software disclaimer: This is the underlying code for the xrhms written as part of the X-ray Histology Project https://www.xrayhistology.org/ funded by the Wellcome Trust (grant 212940/Z/18/Z and WT109682MA) and the University of Southampton. The X-ray Histology facility is located at the University Hospital Southampton and sits within the University of Southampton µ-VIS X-ray Imaging Centre https://muvis.org and the Biomedical Imaging Unit https://www.southampton.ac.uk/biu/.

Please note that this version of the code has had some third-party confidential IP supplied to us under NDA by hardware manufacturers removed from it. While efforts have been made to minimize the impact on the main functionality of the code, we strongly recommend users to perform their own thorough bug-check and edits before attempting to run the code. This will help ensure that any potential issues related to the removal of the confidential IP are addressed.

XRHMS is built using Python 3 and uses the django web framework https://www.djangoproject.com/.

## References

[1] Scott AE Vasilescu DM Seal KA : Three dimensional imaging of paraffin embedded human lung tissue samples by micro-computed tomography. *PLoS One.* 2015;10(6): e0126230. 10.1371/journal.pone.0126230 26030902 PMC4452358

[2] Katsamenis OL Olding M Warner JA : X-ray Micro-Computed Tomography for Nondestructive Three-Dimensional (3D) X-ray Histology. *Am J Pathol.* 2019;189(8):1608–1620. 10.1016/j.ajpath.2019.05.004 31125553 PMC6680277

[3] Lawson MJ Katsamenis OL Chatelet D : Immunofluorescence-guided segmentation of three-dimensional features in micro-computed tomography datasets of human lung tissue. *R Soc Open Sci.* 2021;8(11): 211067. 10.1098/rsos.211067 34737879 PMC8564621

[4] Laundon D Katsamenis OL Thompson J : Correlative multiscale microCT-SBF-SEM imaging of resin-embedded tissue. *Methods Cell Biol.* 2023;177:241–267. 10.1016/bs.mcb.2023.01.014 37451769

[5] Schneider P Katsamenis OL Basford PJ : Foundations for routine 3D X-ray histology (XRH) in Southampton.In: *8 th annual Tomography for Scientific Advancement (ToScA) Global.*Online Edition, Tomography for Scientific Advancement (ToScA), 2020.

[6] Katsamenis OL Olding M Warner JA : X-ray micro-computed tomography for nondestructive three-dimensional (3D) x-ray histology. *Am J Pathol.* 2019;189(8):1608–1620. 10.1016/j.ajpath.2019.05.004 31125553 PMC6680277

[7] Jones MG Fabre A Schneider P : Three-dimensional characterization of fibroblast foci in idiopathic pulmonary fibrosis. *JCI Insight.* 2016;1(5): e86375. 10.1172/jci.insight.86375 27275013 PMC4889020

[8] Xu B Teplov A Ibrahim K : Detection and assessment of capsular invasion, vascular invasion and lymph node metastasis volume in thyroid carcinoma using microCT scanning of paraffin tissue blocks (3D whole block imaging): a proof of concept. *Mod Pathol.* 2020;33(12):2449–2457. 10.1038/s41379-020-0605-1 32616872 PMC7688566

[9] Teplov A Tabata K Fu X : Development of Standard Operating Procedure (SOP) of Micro-computed tomography (micro-CT) in Pathology. *Diagn Pathol.* 2019;5(1). 10.17629/www.diagnosticpathology.eu-2019-5:273

[10] Papazoglou AS Karagiannidis E Liatsos A : Volumetric Tissue Imaging of Surgical Tissue Specimens Using Micro-Computed Tomography: An Emerging Digital Pathology Modality for Nondestructive, Slide-Free Microscopy-Clinical Applications of Digital Pathology in 3 Dimensions. *Am J Clin Pathol.* 2023;159(3):242–254. 10.1093/ajcp/aqac143 36478204

[11] Senter-Zapata M Patel K Bautista PA : The Role of Micro-CT in 3D Histology Imaging. *Pathobiology.* 2016;83(2–3):140–7. 10.1159/000442387 27100885

[12] Navarrete-León C Doherty A Savvidis S : X-ray phase-contrast microtomography of soft tissues using a compact laboratory system with two-directional sensitivity. *Optica.* 2023;10(7):880–887. 10.1364/OPTICA.487270 37841216 PMC10575607

[13] Massimi L Suaris T Hagen CK : Detection of involved margins in breast specimens with X-ray phase-contrast computed tomography. *Sci Rep.* 2021;11(1): 3663. 10.1038/s41598-021-83330-w 33574584 PMC7878478

[14] Rawson SD Maksimcuka J Withers PJ : X-ray computed tomography in life sciences. *BMC Biol.* 2020;18(1): 21. 10.1186/s12915-020-0753-2 32103752 PMC7045626

[15] Metscher BD : MicroCT for comparative morphology: simple staining methods allow high-contrast 3D imaging of diverse non-mineralized animal tissues. *BMC Physiol.* 2009;9: 11. 10.1186/1472-6793-9-11 19545439 PMC2717911

[16] Metscher BD : MicroCT for developmental biology: a versatile tool for high-contrast 3D imaging at histological resolutions. *Dev Dyn.* 2009;238(3):632–40. 10.1002/dvdy.21857 19235724

[17] Walsh CL Tafforeau P Wagner WL : Imaging intact human organs with local resolution of cellular structures using hierarchical phase-contrast tomography. *Nat Methods.* 2021;18(12):1532–1541. 10.1038/s41592-021-01317-x 34737453 PMC8648561

[18] Muller M Kimm MA Ferstl S : Nucleus-specific X-ray stain for 3D virtual histology. *Sci Rep.* 2018;8(1): 17855. 10.1038/s41598-018-36067-y 30552357 PMC6294809

[19] de Bournonville S Vangrunderbeeck S Kerckhofs G : Contrast-Enhanced MicroCT for Virtual 3D Anatomical Pathology of Biological Tissues: A Literature Review. *Contrast Media Mol Imaging.* 2019;2019: 8617406. 10.1155/2019/8617406 30944550 PMC6421764

[20] Withers PJ Bouman C Carmignato S : X-ray computed tomography. *Nat Rev Methods Primers.* 2021;1(1): 18. 10.1038/s43586-021-00015-4

[21] Chen GH Zambelli J Bevins N : X-ray phase sensitive imaging methods: basic physical principles and potential medical applications. *Curr Med Imaging Rev.* 2010;6(2):90–99. 10.2174/157340510791268533 23970846 PMC3747977

[22] Burvall A Lundström U Takman PA : Phase retrieval in X-ray phase-contrast imaging suitable for tomography. *Opt Express.* 2011;19(11):10359–76. 10.1364/OE.19.010359 21643293

[23] Zdora MC Thibault P Kuo W : X-ray phase tomography with near-field speckles for three-dimensional virtual histology. *Optica.* 2020;7(9):1221–1227. 10.1364/OPTICA.399421

[24] Broche L Pisa P Porra L : Individual Airway Closure Characterized In Vivo by Phase-Contrast CT Imaging in Injured Rabbit Lung. *Crit Care Med.* 2019;47(9):e774–e781. 10.1097/CCM.0000000000003838 31162202

[25] Norvik C Westöö CK Peruzzi N : Synchrotron-based phase-contrast micro-CT as a tool for understanding pulmonary vascular pathobiology and the 3-D microanatomy of alveolar capillary dysplasia. *Am J Physiol Lung Cell Mol Physiol.* 2020;318(1):L65–L75. 10.1152/ajplung.00103.2019 31596108

[26] Zeller-Plumhoff B Roose T Katsamenis OL : Phase contrast synchrotron radiation computed tomography of muscle spindles in the mouse soleus muscle. *J Anat.* 2017;230(6):859–865. 10.1111/joa.12606 28369928 PMC5442147

[27] Schulz G Weitkamp T Zanette I : High-resolution tomographic imaging of a human cerebellum: comparison of absorption and grating-based phase contrast. *J R Soc Interface.* 2010;7(53):1665–76. 10.1098/rsif.2010.0281 20659930 PMC2988270

[28] Zanette I Weitkamp T Le Duc G : X-ray grating-based phase tomography for 3D histology. *RSC Adv.* 2013;3(43):19816–19819. 10.1039/c3ra41372a

[29] Ho EML Katsamenis OL Thomas G : 3D X-ray histology for detection of metastasis in whole lymph node specimens. Presented at the 7th Digital Pathology & AI Congress: Europe (03/12/20 - 04/12/20). 2020. Reference Source

[30] Robinson SK Ramsden JJ Warner J : Correlative 3D Imaging and Microfluidic Modelling of Human Pulmonary Lymphatics using Immunohistochemistry and High-resolution μCT. *Sci Rep.* OriginalPaper2019;9(1): 6415. 10.1038/s41598-019-42794-7 31015547 PMC6478691

[31] Lewis RM Pearson-Farr JE : Multiscale three-dimensional imaging of the placenta. *Placenta.* 2020;102:55–60. 10.1016/j.placenta.2020.01.016 33218580

[32] Karavasili C Andreadis DA Katsamenis OL : Synergistic Antitumor Potency of a Self-Assembling Peptide Hydrogel for the Local Co-delivery of Doxorubicin and Curcumin in the Treatment of Head and Neck Cancer. *Mol Pharm.* 2019;16(6):2326–2341. 10.1021/acs.molpharmaceut.8b01221 31026168

[33] Williams KA Gostling NJ Oreffo ROC : Ontogenetic changes in cortical bone vascular microstructure in the domestic duck ( *Anas platyrhynchos*) and ring-necked pheasant ( *Phasianus colchicus*). *J Anat.* 2022;241(6):1371–1386. 10.1111/joa.13741 36000871 PMC9644950

[34] Hough K Anderson L Katsamenis Orestis L : Corroborating µCT and histological data to provide novel insight into the biological response to cochlear implantation at the electrode-tissue interface. Presented at the Tomography for Scientific Advancement - 10th Edition (03/09/20),2020. Reference Source

[35] Zink FE : X-ray tubes. *Radiographics.* 1997;17(5):1259–1268. 10.1148/radiographics.17.5.9308113 9308113

[36] Grant EJ Posada CM Castano CH : A Monte Carlo simulation study of a flat-panel X-ray source. *Appl Radiat Isot.* 2012;70(8):1658–66. 10.1016/j.apradiso.2012.04.011 22738842

[37] Katsamenis OL Burson-Thomas CB Basford PJ : The Reconstruction of Human Fingerprints From High-Resolution Computed Tomography Data: Feasibility Study and Associated Ethical Issues. *J Med Internet Res.* 2022;24(11): e38650. 10.2196/38650 36416872 PMC9730206

[38] Basford PJ Katsamenis OL Boardman R : Integration of a hybrid photon counting detector into a lab-based μCT scanner for 3D X-ray histology. 2021. Reference Source

[39] Willemink MJ Persson M Pourmorteza A : Photon-counting CT: Technical Principles and Clinical Prospects. *Radiology.* 2018;289(2):293–312. 10.1148/radiol.2018172656 30179101

[40] Leng S Bruesewitz M Tao S : Photon-counting Detector CT: System Design and Clinical Applications of an Emerging Technology. *Radiographics.* 2019;39(3):729–743. 10.1148/rg.2019180115 31059394 PMC6542627

[41] Katsamenis OL : UoS 3D X-ray Histology scanner autoloader. ed. YouTube,2021. Reference Source

[42] Schindelin J Arganda-Carreras I Frise E : Fiji: an open-source platform for biological-image analysis. *Nat Methods.* Reviews,2012;9(7):676–82. 10.1038/nmeth.2019 22743772 PMC3855844

[43] Yushkevich PA Gerig G : ITK-SNAP: An Intractive Medical Image Segmentation Tool to Meet the Need for Expert-Guided Segmentation of Complex Medical Images. *IEEE Pulse.* 2017;8(4):54–57. 10.1109/MPUL.2017.2701493 28715317

[44] Koo HK Vasilescu DM Booth S : Small airways disease in mild and moderate chronic obstructive pulmonary disease: a cross-sectional study. *Lancet Respir Med.* 2018;6(8):591–602. 10.1016/S2213-2600(18)30196-6 30072106

[45] Tanabe N McDonough JE Vasilescu DM : Pathology of Idiopathic Pulmonary Fibrosis Assessed by a Combination of Microcomputed Tomography, Histology, and Immunohistochemistry. *Am J Pathol.* 2020;190(12):2427–2435. 10.1016/j.ajpath.2020.09.001 32919981

[46] Boardman R Sinclair I Cox S : Storage and sharing of large 3D imaging datasets.In: *International Conference on 3D Materials Science. *July ed,2012. Reference Source

[47] Scott M Boardman RP Reed PA : Managing heterogeneous datasets. *INFORM SYST.* 2014;44:34–53. 10.1016/J.IS.2014.03.004

[48] Wollatz L Scott M Johnston SJ : Curation of image data for medical research. *Proceedings - IEEE 14th International Conference on eScience, e-Science. * 2018;105–113. 10.1109/eScience.2018.00026

[49] Heinle CE Gaultier NPE Miller D : MetaLIMS, a simple open-source laboratory information management system for small metagenomic labs. *GigaScience.* 2017;6(6):1–6. 10.1093/gigascience/gix025 28430964 PMC5449644

[50] Bendou H Sizani L Reid T : Baobab Laboratory Information Management System: Development of an Open-Source Laboratory Information Management System for Biobanking. *Biopreserv Biobank.* 2017;15(2):116–120. 10.1089/bio.2017.0014 28375759 PMC5397207

[51] Cheng X Cheng K He L : OPENLIMS: The Internet of Things Oriented Laboratory Information Management System. In: *2010 International Conference of Information Science and Management Engineering. *7-8 Aug,2010;1:426–429. 10.1109/ISME.2010.279

[52] Marcus DS Olsen T Ramaratnam M : XNAT: A Software Framework for Managing Neuroimaging Laboratory Data. In: *11th Annual Meeting of the Organization for Human Brain Mapping. *Toronto, Canada,2005.

[53] O'Brien N James K Katsamenis OL : Multi-modal research imaging data management at University Hospital Southampton. presented at the Tomography for Scientific Advancement (11/09/19 - 13/09/19),2019. Reference Source

[54] Basford PJ Konstantinopoulou E Katsamenis OL : A sample and data management system for μCT-based X-ray histology. In: *8 th annual Tomography for Scientific Advancement ( ToScA ) Global. *Online Edition, Tomography for Scientific Advancement (ToScA),2020. Reference Source

[55] Konstantinopoulou E Basford PJ Katsamenis OL : Automated PDF reporting system for X-ray histology (XRH) scan data. In: *8 th annual Tomography for Scientific Advancement ( ToScA ) Global. *Online Edition, Tomography for Scientific Advancement (ToScA),2020. Reference Source

[56] Wollatz L Johnston SJ Lackie PM : 3D Histopathology-a Lung Tissue Segmentation Workflow for Microfocus X-ray-Computed Tomography Scans. *J Digit Imaging.* 2017;30(6):772–781. 10.1007/s10278-017-9966-5 28342044 PMC5681467

[57] Currie HAL Flores Martin N Espindola Garcia G : A mechanical approach to understanding the impact of the nematode *Anguillicoloides crassus* on the European eel swimbladder. *J Exp Biol.* 2020;223(Pt 17): jeb219808. 10.1242/jeb.219808 32748794

[58] Rossides C Pender SLF Schneider P : 3D cyclorama for digital unrolling and visualisation of deformed tubes. *Sci Rep.* 2021;11(1): 14672. 10.1038/s41598-021-93184-x 34282170 PMC8289852

[59] Tabriz AG Mithu MS Antonijevic MD : 3D printing of LEGO® like designs with tailored release profiles for treatment of sleep disorder. *Int J Pharm.* 2023;632: 122574. 10.1016/j.ijpharm.2022.122574 36603670

[60] Garman EF Weik M : X-ray radiation damage to biological samples: recent progress. *J Synchrotron Rad.* 2019;26(4):907–1399. 10.1107/S1600577519009408 31274412

[61] Layton C Bancroft JD Suvarna SK : 4 - Fixation of tissues.In: *Bancroft's Theory and Practice of Histological Techniques (Eighth Edition).* S. K. Suvarna, C. Layton, and J. D. Bancroft Eds.: Elsevier,2019;40–63.

[62] Wolfe D : 6 - Tissue processing.In: *Bancroft's Theory and Practice of Histological Techniques (Eighth Edition).* S. K. Suvarna, C. Layton, and J. D. Bancroft Eds.: Elsevier,2019;73–83.

[63] Eyre DR Paz MA Gallop PM : Cross-linking in collagen and elastin. *Annu Rev Biochem.* 1984;53:717–48. 10.1146/annurev.bi.53.070184.003441 6148038

[64] Zeugolis DI Paul GR Attenburrow G : Cross-linking of extruded collagen fibers--a biomimetic three-dimensional scaffold for tissue engineering applications. *J Biomed Mater Res A.* 2009;89(4):895–908. 10.1002/jbm.a.32031 18465819

[65] Chen CH Hsu MY Jiang RS : Shrinkage of head and neck cancer specimens after formalin fixation. *J Chin Med Assoc.* 2012;75(3):109–13. 10.1016/j.jcma.2012.02.006 22440268

[66] Folk M Heber G Koziol Q : An overview of the HDF5 technology suite and its applications. 2011;36–47. 10.1145/1966895.1966900

[67] Moore J Allan C Besson S : OME-NGFF: a next-generation file format for expanding bioimaging data-access strategies. *Nat Methods.* 2021;18(12):1496–1498. 10.1038/s41592-021-01326-w 34845388 PMC8648559

[68] Moore J Moore W Besson S : ome/ome-zarr-py. 2021. 10.5281/ZENODO.5634522

[69] Pape C Meechan K Moreva E : MoBIE: a Fiji plugin for sharing and exploration of multi-modal cloud-hosted big image data. *Nat Methods.* 2023;20(4):475–476. 10.1038/s41592-023-01776-4 36765247

[70] Boergens KM Berning M Bocklisch T : webKnossos: efficient online 3D data annotation for connectomics. *Nat Methods.* 2017;14(7):691–694. 10.1038/nmeth.4331 28604722

[71] Wilkinson MD Dumontier M Aalbersberg IJ : The FAIR Guiding Principles for scientific data management and stewardship. *Sci Data.* 2016;3: 160018. 10.1038/sdata.2016.18 26978244 PMC4792175

[72] Hall CJ : Diffraction enhanced x-ray imaging of biological tissues.Elettra Synchrotron Light Laboratory, Trieste, Elettra Highlights 2000-2001,2000. Reference Source

[73] Great Ormond Street Hospital for Children NHS Foundation Trust: Helping parents find answers after miscarriage. (Accessed 2022, Nov 15. Reference Source

[74] Docter D Dawood Y Jacobs K : Microfocus computed tomography for fetal postmortem imaging: an overview. *Pediatr Radiol.* 2023;53(4):632–639. 10.1007/s00247-022-05517-1 36169668 PMC10027643

[75] Dawood Y Strijkers GJ Limpens J : Novel imaging techniques to study postmortem human fetal anatomy: a systematic review on microfocus-CT and ultra-high-field MRI. *Eur Radiol.* 2020;30(4):2280–2292. 10.1007/s00330-019-06543-8 31834508 PMC7062658

[76] de Bakker BS de Jong KH Hagoort J : Towards a 3-dimensional atlas of the developing human embryo: the Amsterdam experience. *Reprod Toxicol.* 2012;34(2):225–36. 10.1016/j.reprotox.2012.05.087 22640940

[77] Shelmerdine SC Simcock IC Hutchinson JC : Postmortem microfocus computed tomography for noninvasive autopsies: experience in >250 human fetuses. *Am J Obstet Gynecol.* 2021;224(1):103. e1–103. e15. 10.1016/j.ajog.2020.07.019 32682860 PMC7805479

[78] Lewis C Simcock IC Arthurs OJ : Improving uptake of perinatal autopsy. *Curr Opin Obstet Gynecol.* 2021;33(2):129–134. 10.1097/GCO.0000000000000691 33620891

[79] Simcock IC Shelmerdine SC Hutchinson JC : Human fetal whole-body postmortem microfocus computed tomographic imaging. *Nat Protoc.* 2021;16(5):2594–2614. 10.1038/s41596-021-00512-6 33854254

[80] Katsamenis OL Basford PJ Robinson SK : A high-throughput 3D X-ray histology facility for biomedical research and preclinical applications - Supplementary Data.[Data set]. In: *Wellcome Open Res.* (Version 2). Zenodo. 2023. 10.5281/zenodo.10185486 PMC10620852 37928208

