# Supplementary material for: A high-throughput 3D X-ray histology facility for biomedical research and preclinical applications
Source: Wellcome Open Res. 2023 Dec 8;8:366. Originally published 2023 Aug 25. [Version 2] doi: 10.12688/wellcomeopenres.19666.2 (PMC10620852; doi:10.12688/wellcomeopenres.19666.2)
Supplement: Peer review history

# Reviewer report: Reviewer response for version 1

**DOI:** 10.21956/wellcomeopenres.21783.r66179

**Published:** 2023-11-13

**Authors:** Greet Kerckhofs

**Affiliations:** 1 Institute of Mechanics, Materials and Civil Engineering (IMMC), Catholic University of Louvain, Louvain-la-Neuve, Walloon Region, Belgium

This is a very well-written article clearly describing the functioning of an imaging and image processing/analysis platform for 3D x-ray-based histology of biological tissues. They tackle all the different aspects of the protocol from sample receival until final report writing and data sharing/storage; they also provide very useful guidelines for others to start up or organize their own 3D X-ray-based histology platform. This makes this paper definitely relevant for the community.

 Some remarks on how to further improve the paper:

- The authors mention very often that they are unique, pioneering, etc, but in many of the statements where they mention this, it is actually not the case. The authors are not the inventors of 3D X-ray-based histology, and many other researchers and labs have been working on this technique before. I would suggest to leave out statements as 'it stands out as the world's first XRH facility', 'a previously inaccessible view of soft tissues', 'pioneering', etc. Without these claims, the paper will not be less informative and impactful.
- The authors refer to their methodology (imaging of paraffin-embedded samples using µCT) as 3D X-ray-based histology, and this is correct, but there are also other methods to achieve 3D X-ray-based histology, such as contrast-enhanced µCT imaging. The authors did not refer to this technique, and it is important to mention that their imaging methodology is one way to perform 3D X-ray-based histology, and not the only way. Also cryogenic CECT imaging is a recent technique allowing 3D X-ray-based histology. Finally, another way is phase contrast imaging (using both lab-based µCT devices (recent developments) and sunchrotron facilities). All these methods should be mentioned when the context and state of the art is described.
- The authors mention that their sample preparation and imaging protocol is non-destructive, but the sample preparation processes needed before paraffin embedding and sometimes also the paraffin emebedding itself (can) induce important tissue shrinkage. Hence, the methodology (combining sample preparation and imaging) cannot be referred to as completely non-destructive. Please rephrase, describe in detail the sample preparation process (and the chemical products used), and mention the (potential) influence of the sample preparation steps on the tissue integrity.
- The authors do not refer to the imaging of mineralized tissues (or calcifications). Is this feasible with their methodology? If yes, I would propose to highlight this, as it is an advantage compared to classical 2D histology. Is the imaging of lipids possible? For paraffin embedding, often ethanol or alcohols are used, and they remove the lipids. If this is also the case here, it should be mentioned as a potential limitation.
- A list of advantages and limitations of the methodology would be highly useful. Currently, only the added value is mentioned, but not a lot of attention is given to the current limitations.
- The reviewer highly appreciates the incorporation of the questionnaire and its outcome. Concerning the importance of various aspects, in the text is seemed as if the people had found the absence of a contrast agent as important as having high contrast in the images. However, from Fig. 2, this aspect was shown as being less important (even almost as important as high-throughput data processing). Perhaps adapt this slightly in the text.
- In the description of µCT imaging, the author mentions that radiographs map density variations, but the attuation of a material is not only dependent on the density, but also the atomic number. Further on, the author mentions that (µ) uses X-ray absorption for contrast. As attenuation of X-rays concerning not only X-ray absorption but also scattering, I would propose to explain what the materials' inherent attenuation coefficient is, and then refer to attenuation in the text.
- It would be interesting if the authors would also (briefly) comment on the requirements in terms of required spatial resolution (in the design or selection of a µCT device). What is the minimal resolution proposed? Or what would be guidelines for researchers buying a new µCT device?
- It would be interesting to provide information of the time needed for imaging and image analysis. Of course, this is sample dependent, but an indication of both (or a ratio of both) would be very informative.
- As the authors show that paraffin embedding allows improved contrast between different tissue constituents, I miss a clear explanation why the contrast is increased. Which step/part of the sample preparation will induce the contrast differences? what are the mechanisms behind the differential imaging of tissue constituents? What can they visualize (which differences) and what not? How 'specific' are the contrast differences? Is this allowing to differentiate different proteins from each other? Or is it merely the visualization of pore space versus soft tissue? More information on this would be very useful.
- For Case Study 1, how did the authors validate that the difference between the necrotic region and the healthy region? Based on what can they differentiate? Why would the density of a necrotic region be different from that of a healthy region? Do the authors refer to the volume fraction of tissue as density, or to a change in the X-ray attenuation?
- In the images (for example Fig.10), it would be highly informative to indicate what the reader should focus on, or to indicate what is what. Not all readers are experts in the tissues that have been studied.
- For Fig.11, it would have been useful to also add µCT sections of the samples.
- The reviewer appreciates the sections on data management and storage, which are currently bottlenecks in the entire process.
- In the conclusions, the authors mention the very relevant and highly interesting application of foetal (post-mortem) imaging. The authors mention again that they have pioneered in this, but there are other groups that have been working on this topic already for many years; for example https://www.amsterdamumc.org/en/research/researchers/bernadette-de-bakker.htm. Therefore, be careful with the wording of 'pioneering' or 'being unique', or 'unparalleled'. Please avoid these wordings.

Is the rationale for developing the new method (or application) clearly explained?

Yes

Is the description of the method technically sound?

Yes

Are the conclusions about the method and its performance adequately supported by the findings presented in the article?

Partly

If any results are presented, are all the source data underlying the results available to ensure full reproducibility?

Yes

Are sufficient details provided to allow replication of the method development and its use by others?

Partly

Reviewer Expertise:

3D X-ray based histology using CECT and cryoCECT imaging; tissue engineering; materials characterization; microCT imaging; mechanical testing; 4D-microCT

I confirm that I have read this submission and believe that I have an appropriate level of expertise to confirm that it is of an acceptable scientific standard, however I have significant reservations, as outlined above.

# Response

**Published:** 2023-11-22

**Authors:** Orestis Katsamenis

**Affiliations:** µ-VIS X-ray Imaging Centre, University of Southampton, Southampton, England, UK

Thank you for your thoughtful feedback on our paper. We appreciate your careful consideration and agree that is a few occasions, a more precision in language would be beneficial. Please see below our responses to your comments:

**In response to your 1 ^st^ comment**: we have made revisions to better align our statements with the specific context of our work. We acknowledge that 3D X-ray-based histology is a field with contributions from various researchers and labs. We have focused on clearly articulating the uniqueness of our approach within the defined scope of this paper. We have clarified that, in the context of this paper, 3D XRH refers to microfocus computed tomography-based imaging using conventional, attenuation-based μCT for imaging standard unstained FFPE blocks in a lab environment. We would like to emphasise that the term, as used in this paper, encompasses a complete and dedicated workflow for 3D XRH, which is seamlessly integratable with clinical workflows and is not disruptive to them. Nevertheless, we recognise and wholeheartedly support the idea that the term '3D X-ray Histology’ should be used to cover a much wider spectrum of approaches and modalities, including phase, contrast, and SR CT. Regarding our comment about it being “the world's first XRH facility”, please allows us to respectfully stand by our statement, and provide further clarification. In our understanding, the XRH facility in Southampton is indeed a unique entity globally. It is designed with the explicit purpose of providing X-ray histology imaging services and supporting third-party research initiatives. That is not to say the XRH facility team is the first to conduct micro-CT-based histology research, far from it. While we acknowledge that 3D histology work and research have been conducted by various researchers and labs worldwide, our claim specifically relates to the dedicated infrastructure we have implemented. To clarify, the uniqueness of our facility lies in its structured setup, with the intention of facilitating broader access and utilisation by external users including the NHS.

**In response to your 2 ^nd^ comment**: That is a valid comment and one raised by other reviewers. We want to emphasise that, given the comprehensive nature of the information to be included in our manuscript, we made a deliberate decision not to expand into an exhaustive literature review encompassing all the diverse methods of X-ray CT for histology applications. Instead, we have chosen to present the initial stages of our research journey, which have culminated in the development of what we term as 3D X-ray Histology. We have explicitly addressed this choice at the conclusion of the first paragraph in the introduction of our manuscript, where we encourage readers to explore additional published articles, including those discussing phase-/contrast-enhanced µCT imaging.

**In response to your 3 ^nd^ comment**: Our primary focus when claiming a non-destructive / non-intrusive nature of our X-ray-based histology technique is within the context of classical histology and after the formalin-fixed paraffin-embedded (FFPE) stage. Preceding stages, including sample preparation and paraffin embedding, are elements that fall outside the direct control of 3D XRH, as our approach is designed to handle specimens that have undergone the classical preparation journey. However, we appreciate your observation that classical tissue handling, including the processes leading to FFPE, can affect tissues and introduce disturbances such as tissue shrinkage. These inherent challenges are now acknowledged. Going forward, we have included additional details in the Conclusions paragraph to clarify what’s been discussed here. Please refer to the segment beginning with “ *At this point, it is important to note that, while the non-destructive/non-intrusive nature of XRH imaging holds true for* […]” to “ […] *use relative measures and their study sample groups and controls have undergone the same level of processing*”

**In response to your 4 ^th^ comment: **We intentionally omitted it from our discussion for two key reasons. Firstly, micro-CT has been a well-established tool for imaging mineralised tissues over several decades, with documented and proven performance across different scales. Our emphasis has been on addressing the challenges associated with imaging soft tissues, arising from them exhibiting minimal X-ray contrast; a long-standing issue in the field. Secondly, in classical histology, mineralised tissues typically undergo demineralisation before further processing. As a result, even histological samples like bone marrow biopsies would have undergone demineralisation by the time they reach the FFPE stage, which is considered our “entry point”. There are, of course, FFPE specimens that have mineralisation that is not expected and we successfully image them with slight alterations in the imaging protocol. With regards to lipid imaging, we have recently undertaken work where visualising lipids is exactly what the XRH facility was commissioned for. Although you are correct that FFPE processing strips lipids from tissues, this has been advantageous in the project we have undertaken as “casts” form and thus can be analysed for volumetric and spatial information. It is also compatible with correlative imaging approaches as biomarkers are retained on the cell surfaces of adipocytes and thus can be confirmed as lipid casts post-scanning. This application of XRH for this form of imaging is also under preparation for publication.

**In response to your 5 ^th^ comment: **As we image a vast number of samples for a wide range of research projects, it is difficult to give an overarching list of advantages and disadvantages to the generic method, as the methods vary depending on the sample being imaged and other project-specific constrains. For example, shrinkage due to FFPE processing is a disadvantage to some research workflows (e.g. image-based modelling) where absolute length scales are critical to the question. However, in a clinical project where the presence or absence of a diagnostic feature is of interest, the overall shrinkage of said tissue is not considered relevant for evaluation.  However, following your recommendation, we have outlined some of the more-prominent limitations associated with the generic facility workflow, which are now added to our manuscript:

1. **Imaging Trade-offs:** Balancing the trade-off between field of view and resolution, as well as resolution to scanning time (and thus cost), poses a challenge that needs to be carefully considered.
2. **Data Size Challenges:** The size of data generated poses challenges for curation, archiving, and open-data strategies, particularly in studies requiring high *n* numbers. Additionally, ambiguity regarding what constitutes raw data (raw uncorrected collected radiographs, corrected radiographs, float-point reconstructions, processed 16-bit / 8-bit volumes…?) introduces an extra layer of complexity.
3. **Complexity of Image Analysis:** Managing the intricacies of image analysis, particularly in terms of quantification, presents a significant challenge to many users, especially the ones with no or limited experience to processing of volume data.
4. **Need for Correlative Imaging:** Correlative imaging is often required for biomarker identification, which adds an additional layer of complexity (and cost) to the workflow.
5. **Contrast Challenges with Non-Stained Soft Tissues:** The majority of non-stained soft tissues, when imaged in a hydrated state exhibit minimal X-ray contrast. For such specimens other approaches are much better suited, and should be considered including phase-contrast imaging, contrast CT and SRCT.
6. **Cost of Relevant Equipment:** The acquisition and maintenance costs associated with the necessary equipment capable of handling XRH data, including software and network infrastructure, can be substantial and lead to underutilisation of the data. For this reason it is important that an XRH facility should be prepared to invest and offer access to processing infrastructure.
7. **Standardisation and DICOM Compatibility:** Ensuring standardisation and compatibility with DICOM (Digital Imaging and Communications in Medicine) standards remains a crucial consideration, and one that can unlock clinical compatibility.

**In response to your 6 ^th^ comment: **While we had explicitly mentioned, in accordance with Figure 2, that "High image contrast from unstained tissues [...] was highlighted as being of high importance by **approximately half of the participants**", we have revised our statement to further mitigate the possibility of misinterpretation. We would like once again to emphasise that our findings should be understood within the context of the classical histology workflow. Through this lens, staining prior to sectioning may be perceived as an intrusive or destructive process, and, in some instances, can pose challenges for administrative or practical reasons, particularly when dealing with archival tissue or clinical specimens.

**In response to your 7 ^th^ comment: **The reviewer's observation is indeed accurate. In the relevant section of the text, our intention was to provide a simplified, layperson's description of the basic principles of the technique, aiming to make the content accessible without delving into highly technical details. However, we recognise the importance of ensuring accuracy in the representation of the underlying physics. We have now modify our text accordingly to better convey the complexity of the physics involved, clarifying that the attenuation of X-rays is influenced by other factors beyond material density as well, and we have included a reference to a paper by Withers et al for readers interested in a more in-depth understanding.

**In response to your 8 ^th^ comment:** In response to comments raised by another reviewer we have incorporated comprehensive resolution characterisation data into the supplementary materials (see https://doi.org/10.5281/zenodo.10185486 ). Based on an extensive dataset exceeding 2,500 scans recorded in our system, we can provide details on the mean voxel size, which is 0.021 mm ± 0.023 mm. The median value is 0.012 mm, with minimum and maximum values of 0.001 mm and 0.180 mm, respectively. Our experience indicates that, for micro-anatomy work, a resolution within the range of 5 to 15 µm proves sufficient for the majority of applications. However, it is worth noting that more specialised applications, such as correlative proteomics or genomics, may necessitate higher resolutions.

**In response to your 9 ^th^ comment: **Unfortunately, providing a specific indication or ratio for both imaging and image analysis is challenging due to the highly sample-dependent nature of these processes, which you rightly pointed out. The balance between imaging and analysis time is influenced by factors such as the qualitative versus quantitative nature of the imaging. In general, the time investment for quantitative imaging tends to lean heavily towards the processing stage rather than the acquisition. In the manuscript, we have included a reference to the "most common" imaging protocol for FFPE samples in the part where we are discussing the risk of radiation damage, indicating acquisition times typically ranging from 2 to 5 hours. However, how users handle the data downstream varies significantly across applications, making it challenging to be more specific or provide a standardised ratio for imaging and analysis times.  

**In response to your 10 ^th^ comment: **In the realm of soft-tissue imaging, particularly within the context of Formalin-Fixed Paraffin-Embedded (FFPE) tissue, the majority of components consist of organic material with comparable attenuation coefficients. Our non-staining protocol captures minute X-ray attenuation variations introduced by the FFPE processing. The impact of FFPE on tissue physical properties is well-documented, and we could extrapolate the physical effects into the X-ray imaging domain. Formaldehyde, a key component, facilitates the cross-linking of native tertiary protein structures through aldehyde groups within the tissue. This process increases electron density in the cross-linked regions, consequently affecting their X-ray attenuation properties. Regions with heightened cross-linking appear "denser" than their non- or less-cross-linked counterparts. The FFPE process also induces tissue dehydration, resulting in both global and local tissue shrinkage and densification. The dehydration steps in FFPE play a pivotal role in increasing the molecular density per unit space. This is primarily attributed to the removal of substantial quantities of hydrogen and oxygen elements from the sample, leading to a densification of the organic elements proportionate to their hydration level. This densification contributes to subtle distinctions between elements with varying degrees of hydration.   These subtle attenuation differences resulting from the FFPE process is what we exploit for contrast. Our intention in future is to systematically gather specific data elucidating the precise contributions of each processing stage to the enhanced contrast in the sample. Furthermore, we aim to investigate the impact of different forms of commonly used fixatives in histological protocols on contrast levels. This comprehensive approach will provide a deeper understanding of the intricate relationships between tissue processing, physical properties, and contrast enhancement, thereby refining our soft-tissue imaging techniques. At this stage we cannot in confidence comment on the specificity of these effects. For cases where specific imaging is crucial, we incorporate correlative conventional histology and immunohistochemistry.   The above has now been included in our closing remarks in the Conclusions paragraph. Please refer to the segment beginning with “ *At this point, it is important to note that, while the non-destructive/non-intrusive nature of XRH imaging holds true for* […]” to “ […] *use relative measures and their study sample groups and controls have undergone the same level of processing*”

**In response to your 13 ^th^ comment:** Thank you for your comment. The source publication is freely accessible to all, and we believe it would be more appropriate for any interested party to access the link for a comprehensive understanding of the entire work. In addressing your feedback, we have now included a reference to the pertinent figure in the source publication, encouraging readers to explore it for further details.

**In response to your 14 ^th^ comment**: Thank you for your kind words!

**In response to your 15 ^th^ comment**: We would like to clarify that in our manuscript, when we referred to pioneering efforts, we were specifically highlighting the work conducted by the Great Ormond Street Childrens' Hospital (GOSH) team, led by Dr. Owen Arthurs in London, UK. Our intention was to underscore the clinical nature of foetal post-mortem imaging and the extensive development and validation work carried out by the GOSH team that allowed it to be deployed in a clinical setting and offered as a clinical service. The clinical service at GOSH includes a Nikon Med-X scanner incorporating the same enhancements prototyped in Southampton. In response to your comment and to enhance accuracy, we have incorporated the work of Dr. Bernadette De Bakker's group at Amsterdam UMC and have specified the significance of the GOSH team within the UK landscape.

# Response

**Published:** 2023-11-22

**Authors:** Orestis Katsamenis

**Affiliations:** µ-VIS X-ray Imaging Centre, University of Southampton, Southampton, England, UK

**In response to your 11 ^th^ comment: **Please note that case-study 1 is based on a previously published work. The authors did not specifically conduct this study for the present research; instead, it is referred to as an exemplar. Notably, the only author from the current list involved in the cited work is Dr. Katsamenis. In the referenced publication, necrotic areas were identified by clinical pathologists using conventional histology slices taken after XRH imaging from the same specimens. Subsequently, these identified necrotic areas were cross-correlated with the XRH data (cf. Figure 8 in doi.org/10.1021/acs.molpharmaceut.8b01221 ) and segmented in 3D. These areas had a characteristically high Calcium content and appeared brighter in CT. Volume fraction was calculated as the ratio between the total tumour volume and the necrotic volume.

**In response to your 12 ^th^ comment:** Thank you for the recommendation. We will investigate the options available at this stage of the submission.

# Reviewer report: Reviewer response for version 1

**DOI:** 10.21956/wellcomeopenres.21783.r68679

**Published:** 2023-10-31

**Authors:** Chulong Zhang

**Affiliations:** 1 Chinese Academy of Sciences, Shenzhen Institute of Advanced Technology, Shenzhen, China

This paper reports an important high-throughput 3D X-ray histology facility. It is indeed remarkable and has rendered many contributions. Here exist only minor concerns and comments:

1. Would it be possible to briefly expand on the principles and methods of 3D X-ray Histology (XRH) at the beginning of the paper?
2. In Case Study 2, how were the 2D histological slices registered with the 3D XRH? Or, was a 2D slice of the XRH used?
3. In Case Study 3, could you possibly provide some specific data or tables to support your conclusion, or add more explanation? The current text and visual results alone can be somewhat confusing.

Is the rationale for developing the new method (or application) clearly explained?

Yes

Is the description of the method technically sound?

Yes

Are the conclusions about the method and its performance adequately supported by the findings presented in the article?

Yes

If any results are presented, are all the source data underlying the results available to ensure full reproducibility?

Yes

Are sufficient details provided to allow replication of the method development and its use by others?

Yes

Reviewer Expertise:

X-ray, CT, Medical Physcis, Pathology Image Processing

I confirm that I have read this submission and believe that I have an appropriate level of expertise to confirm that it is of an acceptable scientific standard.

# Response

**Published:** 2023-11-22

**Authors:** Orestis Katsamenis

**Affiliations:** µ-VIS X-ray Imaging Centre, University of Southampton, Southampton, England, UK

Thank you for your kind words. Please find our responses below.

**Comment 1**: Thank you for your suggestion. We have incorporated a brief expansion on the principles and methods of 3D XRH at the beginning of the. This section now includes an outline and references for further exploration. We hope that this addition enhances the clarity and understanding of XRH for our readers.

**Comment 2**: The image show in Figure 10 shows a superposition of the light microscopy image onto a single XRH slice illustration purposes. The registration method used requires the H&E histology slice to be elastically registered onto the μCT volume after the latter has been rigidly re-oriented in space so that XY slices run perfectly parallel to the cutting plane of the microtome. The process is detailed in Katsamenis et al ( https://doi.org/10.1016/j.ajpath.2019.05.004 ), and in Lawson et al ( https://doi.org/10.1098/rsos.211067 ).

**Comment 3**: Case study 3 serves as a reference to a prior study carried out using our facility, and it was not conducted explicitly for this manuscript. Detailed information about this work can be found in the original publication by Robinson et al. at https://doi.org/10.5258/SOTON/D0310 . The inclusion of this reference here aims to emphasise the significance of XRH in producing high-fidelity 3D isotropic geometries for precise soft-tissue image-based modelling.

# Reviewer report: Reviewer response for version 1

**DOI:** 10.21956/wellcomeopenres.21783.r69307

**Published:** 2023-10-26

**Authors:** Marco Endrizzi

**Affiliations:** 1 Medical Physics and Biomedical Engineering, University College London, London, England, UK

This paper describes the 3D X-ray histology facility at University Hospital Southampton. The work is presented as an overview of the facility covering motivation, methods and results. Below are some suggestions and comments for the Authors’ consideration.

 Abstract: Background, I suggest including a broader perspective on X-ray virtual histology, as it currently reads rather self-referring. Methods: I suggest highlighting what the novelty elements are, as this is not immediately clear from reading this part. Results: I suggest avoiding subjective metrics like “numerous” and focus instead on more objective results. Conclusion: I would welcome a more comprehensive review of the field in support of these conclusions, or a mitigation of these.

 Introduction, as above, I suggest including a broader perspective on the field, surely much wider than just the proof-of-principle study published in 2015

 “The first complete system for 3D XRH …” this should refer specifically to the UHS facility.

 Med-x prototype: discussion about the choice of metal and accelerating voltage would be valuable here; as well as details about the spatial resolution characterisation (either results or citation). 

 Customised XRH “significant advantages over static reflection target” what are the advantages? How significant are they? ”in ways not previously possible” adding details would help clarifying.

 “provide previously inaccessible options for scanning biological samples” also here adding details would help readers in identifying the improvements.

 DECTRIS detector and spectral capabilities are mentioned but not really discussed, details in this section would help identifying the advantages and constraints of such development.

 Data visualisation: is there a preferred modality/tools from the users? 

 Figure 7 and 9 using panel letters would help referencing to the correct image from caption.

 FFPE is a common acronym but should be defined.

 Client access: a small table showing field of views and spatial resolution achievable would help in understanding the necessary trade-offs.

 In conclusion, I find this is a comprehensive report on a 3D X-ray histology research facility with impressive developments towards making this type of imaging readily accessible to users and clients. The topics covered range from the design of the facility to technical details on the hardware, software tools and case studies. Some points have been identified for the Authors’ consideration where the description and discussion can be made more precise and complete to the benefit of the readers.

Is the rationale for developing the new method (or application) clearly explained?

Yes

Is the description of the method technically sound?

Yes

Are the conclusions about the method and its performance adequately supported by the findings presented in the article?

Yes

If any results are presented, are all the source data underlying the results available to ensure full reproducibility?

Yes

Are sufficient details provided to allow replication of the method development and its use by others?

Yes

Reviewer Expertise:

X-ray imaging

I confirm that I have read this submission and believe that I have an appropriate level of expertise to confirm that it is of an acceptable scientific standard.

# Response

**Published:** 2023-11-22

**Authors:** Orestis Katsamenis

**Affiliations:** µ-VIS X-ray Imaging Centre, University of Southampton, Southampton, England, UK

Thank you for your time to review our manuscript and your feedback. Please see below our responses to your comments:  

**Comment RE Novelty elements**: The first reviewer also brought up this aspect. In the revised version (Version 2) of our manuscript, we have taken steps to emphasise the novel elements and made corresponding updates to the document. As a result, the paragraph “Innovative Hardware Advancements” has been added.  

**Comment RE Broader perspective on the field**: Please note that, due to the already substantial amount of information required for inclusion in our manuscript, we have chosen not to provide an extensive literature review encompassing all the variations of X-ray CT for histology applications. Instead, we have introduced the initial stages of our journey that ultimately led to the development of what we now refer to as *3D X-ray Histology*. In this context, we introduce the XRH facility and outlined the rationale behind our research efforts. That is not to undervalue the extensive work that has been carried out in this field by other colleagues. We have clarified this perspective at the end of the first paragraph in the introduction in our manuscript, and readers are encouraged to refer to published articles for a more comprehensive overview of the field. Nine overview articles are listed covering a range relevant to this work including:

- [11]      M. Senter-Zapata, K. Patel, P. A. Bautista, M. Griffin, J. Michaelson, and Y. Yagi, "The Role of Micro-CT in 3D Histology Imaging," Pathobiology, vol. 83, no. 2-3, pp. 140-7, 2016, doi: 10.1159/000442387.
- [12]      C. Navarrete-León et al., "X-ray phase-contrast microtomography of soft tissues using a compact laboratory system with two-directional sensitivity," Optica, vol. 10, no. 7, 2023, doi: 10.1364/optica.487270.
- [14]      S. D. Rawson, J. Maksimcuka, P. J. Withers, and S. H. Cartmell, "X-ray computed tomography in life sciences," BMC Biol, vol. 18, no. 1, p. 21, Feb 27 2020, doi: 10.1186/s12915-020-0753-2.
- [19]      S. de Bournonville, S. Vangrunderbeeck, and G. Kerckhofs, "Contrast-Enhanced MicroCT for Virtual 3D Anatomical Pathology of Biological Tissues: A Literature Review," Contrast Media Mol Imaging, vol. 2019, p. 8617406, 2019, doi: 10.1155/2019/8617406.

**Comment RE “The first complete system for 3D XRH …”**: We value your comment, but we respectfully stand by our statement. Please allow us to provide further clarification regarding our statement. As we understand it, the Med-X system marked a significant milestone as the first complete attenuation-based (i.e. conventional μCT) system explicitly engineered with histology applications in mind, and it was purposefully designed for installation in clinical and hospital settings. This distinction is a critical aspect of our work, as it underscores the unique and pioneering nature of the Med-X prototype. In addition to being the first of its kind, its influence is evident in the fact that the current commercially available system by 3DHISTECH (PANORAMIC-X) shares a substantial portion of its components with the original Med-X prototype, while the enclosure design has been maintained fully. This not only reaffirms the innovative approach we took in developing the system but also underscores the lasting impact it has had on subsequent developments in the field of 3D X-ray Histology.  

**Comment RE Med-X prototype**: Target metals - The metals listed in our manuscript are the ones designated by Nikon as their 'standard' multilateral reflection targets. We have not altered the assortment of reflection target metals in any way. Each metal has its pros and cons, with each one producing a unique X-ray spectrum possible suited to different applications, but this is something we are still exploring; Resolution – In these systems spatial resolution depends upon a number of factors including, geometrical parameters (focus-to-detector / focus-to-object distances), image quality (e.g. presence of scattering, noise or contrast resolution), and of course X-ray source characteristics. For this reason, in our manuscript we are referring to the “smallest” focal-spot size of the electron beam onto the target, as measure of the highest achievable spatial resolution. Characterisation of that property was achieved using resolutions phantoms (JIMA, QRM) in radiography or tomography modes, although other volumetric methods are also applicable (e.g. DOI 10.1088/1361-6501/aa85b7). We have now updated the supplementary data file to include spot-size characterisation data (see https://doi.org/10.5281/zenodo.10185486 ); The energy range selection is addressed in “Innovative Hardware Advancements”  

**Comment RE Customised XRH**: The Mo rotating target is optimised for high-throughput imaging. The rotation of the target wheel in a rotating target exposes different portions of the target material to the electron beam over time, resulting in improved heat dissipation. This allows the electron beam to maintain its optimal focus for double the power compared to a reflection target. The process of spot-defocusing, which spreads the heat load onto a larger area and safeguards the target, initiates at 14W instead of 7W. Furthermore, the defocusing curve is gentler compared to that of a reflection target, enabling the source to operate at a spot size smaller than 100 μm at 100 W. In practical terms, this leads to shorter scan times for the same resolution or an increase in resolution by over a factor of 2x when using X-ray power exceeding 30W; i.e. a spatial resolution at 30 W of ~ 45 μm and 90 μm for the rotated and the reflection target respectively. – This in now further clarified in the updated version of our paper and supporting data are provide in the accompanying supplementary available via Zenodo; See for example *202107_MoRot-testing_Edge-mod-function-usingQRMrecons.png*.   We have also rephrased the “provide previously inaccessible options for scanning biological samples”. The text now reads: “ *The improvements to the x-ray scanning hardware that have been integrated into both the Med-X and XRH scanners including customised X-ray anodes, auto-loader and ability to mount a DECTRIS SANTIS 3204HR, enrich the user experience by providing a singular, comprehensive suite of cutting-edge equipment specifically tunned for soft-tissue imaging available at a single facility. This resource empowers users with an expanded and diverse range of acquisition modes to choose from.”*  

**Comment RE DECTRIS integration**: This was also raised by Reviewer 1. Please kindly refer to our reply to them.  

**Comment RE Data visualisation**: In our experience, data visualisation tools are often chosen based on individual user preferences and familiarity with specific packages. Our centre provides high-end computational facilities with a diverse array of both open-source and commercial visualisation and processing suites, including ORS Dragonfly, Avizo, VGStudioMax, Simpleware ScanIP, and Fiji/ImageJ. Users typically make their selections considering factors such as the time available for analysis, the necessity for quantification, and whether XRH visualisation is the end goal or a stepping stone for more advanced analysis, such as image-based modelling.  

**Comment RE table showing field of views and resolutions**: We understand your point completely. Initially, we considered creating such a table as well. However, upon careful consideration, we realised that a static table would only provide a partial view of the whole picture due to the numerous degrees of freedom involved. For instance, presenting a "best case" scenario for each parameter, such as the smallest focal spot or maximum field of view, might not accurately represent the system's capabilities. Resolution depends on several factors, including the field of view, detector binning, X-ray power / energy (both of which affects focal spot geometry). Additionally, there are options like vertical stitching, offset tomography, and local tomography that can be used to fine-tune the balance between field of view, resolution, and acquisition time. For these reasons, we've decided against presenting such a table as it could potentially lead inexperienced users to draw incorrect conclusions and misrepresent the hardware's capabilities. Instead, we recommend that users engage in a discussion with the imaging scientist who will be managing their session to tailor the parameters to their specific needs effectively.

# Reviewer report: Reviewer response for version 1

**DOI:** 10.21956/wellcomeopenres.21783.r66178

**Published:** 2023-10-12

**Authors:** Alessandro Olivo

**Affiliations:** 1 Institute of Healthcare Engineering, University College London, London, England, UK

This report provides a thorough description of a unique and important facility that has already benefitted a large number of biomedical researchers and has the potential to benefit more in the future. For these reasons, I have no hesitation in recommending publication of this manuscript in Wellcome Open Research, with only a few optional suggestions.

 I’ll start with an issue of personal taste – to me it sounds a bit weird for authors to say themselves that “The 3D X-ray Histology (XRH) facility at UHS is a pioneering platform…”. I think they could iterate the large number of successful projects they conducted, and they’re surely welcome to say that to their knowledge this is the first such facility etc. On a similar note, I wonder whether the sentence “XRH is a microfocus computed tomography-based technology developed upon the proof-of-principle study published in 20151” should be somewhat mitigated, since several studies proposed to use x-rays for virtual histology, especially at synchrotrons.

 I would welcome some degree of expansion on the subject of hardware improvements of the scanners – are these limited to the availability of multi-target materials and of the optional rotating Mo target? Could these be encountered more widely in available commercial scanners – or, in other words, what is it that makes the MedX and modified 225 “specific” to the imaging of histopathology cassettes?

 On a similar note, could the authors explain better when the MedX and when the 225 is used – what is the choice based on?

 I was impressed with the auto-loader development that makes operations smoother and more cost effective – out of curiosity does filament lifetime then become a bottleneck?

 The DECTRIS photon counter is mentioned but very little is said about how it could improve performance – do the authors have any hint on practical improvements? Does it impose a reduction in specimen size, and if so by how much? Are gaps between detector modules an issue?

 Fig 7 add letters (a-f) to panels, add box in a-c to indicate area where d-f where extracted from. Same for fig 10 (box only).

 Generic artifact processing is mentioned but as well known ring artifacts are often a prominent one in this context. How are these handled? Is jitter used?

 I found the sentence “The standard range of equipment allows imaging at spatial resolution down to 5 μm, although higher resolutions can be achieved with minor modifications of the hardware.” (page 16) intriguing – how would this be achieved (considering the resolution ~ 2 x focal spot rule of thumb), and is it currently available?

 Another interesting part is the discussion as to whether x-rays can affect subsequent sample analysis. If I understood correctly, the authors cite a synchrotron paper that says they do [54], and a lab-based one that says they don’t [7], and seems to attribute this to the different flux ranges – however I would argue that a good quality CT image depends on the total number of collected photons, which (assuming comparable detector technology is used, which seems to be the case) is probably similar in the two cases, and obtained through much longer scans in the lab case. This possibly goes beyond the scope of this paper, but it would be good to see some additional comment from the authors on this important point.

 To possibly end as I started on a matter of personal taste – I would avoid expressions such as “immense potential” (p. 18).

 While I hope the authors will agree with me that integration of at least some of the above comments would make for a more complete report, I remain convinced that it should be published regardless – I have read it with great interest.

Is the rationale for developing the new method (or application) clearly explained?

Yes

Is the description of the method technically sound?

Yes

Are the conclusions about the method and its performance adequately supported by the findings presented in the article?

Yes

If any results are presented, are all the source data underlying the results available to ensure full reproducibility?

Yes

Are sufficient details provided to allow replication of the method development and its use by others?

Yes

Reviewer Expertise:

x-ray detectors and imaging

I confirm that I have read this submission and believe that I have an appropriate level of expertise to confirm that it is of an acceptable scientific standard.

# Response

**Published:** 2023-11-22

**Authors:** Orestis Katsamenis

**Affiliations:** µ-VIS X-ray Imaging Centre, University of Southampton, Southampton, England, UK

Thank you for your thorough feedback and your kind words. Please see below our responses to your comments:

 We would like to clarify that by saying “ *XRH […] is a pioneering platform*” we are referring to the whole ecosystem we have developed in Southampton, which incorporates hardware advancements, IT infrastructure, data processing and management workflows of the XRH data, and support of correlative imaging workflows with direct integration of conventional histology and electron microscopy. We strongly believe that in this context, our phrasing stands true. We are by no means implying that micro-anatomical characterisation of soft tissue samples using μCT emerged from Southampton. As you correctly pointed out, several studies over the years have proposed (and “proved” we add) that X-rays technology can be used for histological imaging.

 Similarly, our phrase “ *XRH is a microfocus computed tomography-based technology developed upon the proof-of-principle study published in 2015*” was also referring to a technology using conventional, attenuation-based μCT imaging for imaging standard unstained FFPE blocks in a lab-environment. Up to that point, μCT imaging of such specimens under these conditions was considered extremely challenging if not impossible. However, we do agree that XRH is, and should be, a broader term covering other X-ray modalities including SRμCT, phase-contrast imaging, photon-counting, etc. That for we will be revising this phrase as follows:

- " *XRH in the context of this paper represents a pioneering attenuation-based microfocus computed tomography-base (μCT) technology developed as a result of the proof-of-principle study published in 2015. The term extends its applicability to encompass complementary X-ray μCT modalities, such as synchrotron-based μCT, phase-contrast imaging, photon-counting imaging, and more, reflecting its broader scope.*"

**On the subject of hardware improvements**: we have collaborated with our industrial partner over the years to optimise our systems, aiming to achieve the highest image quality possible when imaging standard soft-tissue FFPE tissue cassette specimens. Our focus has also been on ensuring that these improvements are applicable in a clinical setting. Below, we highlight some of the modifications we have made:

- Studied and made an adjustment in the electron angle of attack onto the target with the goal of minimising the heel-effect, which can lead to an uneven mean energy profile of the X-ray beam between the bottom and the top of the illumination field. This is important for quantitative imaging and for the consistent visualisation of the tissue across the whole field of illumination.
- Studied and modified the housing in the X-ray source to significantly reduce the generation of secondary sources resulting from the interaction of back-scattered electrons with its housing. While this phenomenon is negligible and causes no problems when materials with high attenuation contrast are imaged, it was causing issues when we were imaging unstained FFPE specimens. These specimens required us to work within a very tight contrast windows, in which "phantom" images of the specimens resulting from the secondary sources could be seen.
- Modified the enclosure of the Med-X prototype to reduce its footprint and its weight. We did that by reducing the maximum energy of the system to 130kVp by using a soft-limited 160kVp source. We also “dressed” its inner components to allow for easy cleaning and reduce the risk of biohazardous contaminations in a clinical environment.
- We modified the standard W rotating target to a Mo rotating target, to allow for higher flux soft X-ray imaging and improving throughput more clinically relevant time-frames. This was paired with the development and deployment in our XRH XT H 225 ST system of our automatic sample exchanger.
- The sample racks for the autoloader system have been specifically designed to allow either the maximum number of standard 30 mm histology cassettes to be loaded or to provide 10 slots which may contain either a standard 30 mm cassette or a 60 mm giga-cassettes.
- We also modified the standard W transmission target to Mo to allow imaging with softer X-rays at much higher resolution.

A range of these improvements are currently used in the commercially available system (PANORAMIC-X) that was made available to clinical and academic parties for purchasing by 3DHISTECH Ltd in summer 2023.

**Regarding the use of each system**, XRH XT H 225 system is mainly used for high throughput studies requiring multiple specimens to be scanned using the same protocol. The automatic sample exchanger allows us to set up unsupervised multiday sessions. It is also used for studies requiring higher spatial resolution for a larger field of view. That’s possible due to it’s 2850 x 2850 139 μm pixel pitch detector compared to the 2000 x 2000 200 μm pixel pitch used in the Med-X. Both the Molybdenum rotating target and Molybdenum transmission target are only available in the XRH scanner. Other than these, the two system are interchangeable in many aspects and often the system selection is based upon availability, user familiarity and other logistics issues.

 Moving on to the next comment, the reviewer is correct to assume that the **filament lifetime** can become the bottleneck. An average lifetime of a filament at XRH-specific imaging setting ranges from 200 - 400 h. Our autoloader supports up to 14-specimens so for protocols that require imaging for longer than 14 hr / sample filament lifetime can be an issue. In our experience, if the user is keeping track of beam-on time, which we do, and changes the filament before long sessions, the likelihood of it failing within a single session is minimal. Our protocols are also rarely exceeding 6 h / samples with more of them being around 2 -3 h / sample, which means multiple auto loader racks can be scanned using the same filament.

**Regarding the DECTRIS detector**, at this stage, we can only speculate about its benefits and our main effort was focused on developing hardware and software to integrate the detector into our systems, and the raw image processing side of things. As mentioned, the field of view on Y-direction (height) is significantly smaller, so realistically for histological-relevant resolution helical scanning will need to be used, which is doable in our system. The gaps do pose issues, but image post-processing along with acquisition strategies can mitigate these issues. We are in the process of finalising a paper in which we describe all these developments in detail. Our main hope is that this technology will allow us to use a much narrower beam energy profile and k-edge imaging hopefully further improving the contrast and perhaps the specificity of XRH imaging. Dual-energy imaging will also become possible with single acquisitions.

**On the artefact-corrections**: Ring artefacts are handled on the acquisition side by using jittering/shuttling of the specimen across a small range of pixels along the X-direction, which aligns with the detector’s horizontal dimension. The most common artefacts in this context are cassette artefacts arising from beam-hardening, minor scattering, and in cases of dense objects in the tissue, such as calcium, metal or contrasted sutures, beam-starvation.

 The higher resolution mentioned can be achieved using a customised Mo-transmission target. This target supports a spot-size with a diameter in the order of 1.3 μm. With a rule of thumb mentioned achievable spatial resolution is in the order of 2.6 -3 μm. With our 2850 x 2850 dexel detector a specimen with an effective diameter up to 3.5 mm (e.g. a cryo-biopsy or a needle biopsy) can be imaged without resorting to local-tomography.

**Regarding the radiation damage**: The reviewer raises a valid point, and it’s a matter our community should examine more closely. The comment they made is of course correct. What truly counts in terms of deposited dose, assuming all other factors such as photon energy and sample attenuation characteristics remain comparable, is the actual number of photons interacting with the sample during imaging. The key point we intended to emphasise is the dose-dependence of damage. In many cases, researchers turn to high brilliance synchrotron sources (1e12 photons /s ) to surpass the limitations of laboratory equipment and enhance image quality, rather than matching. This often leads to the use of significantly higher dose protocols.    A direct comparison between the two papers referenced in our manuscript in absolute dosimetry terms is challenging for a number of reasons including, beam spectrum profiles (polychromatic vs monochromatic) and tissue condition (fixed/cross-linked vs fresh/hydrated). Nevertheless, based on the limited data available to date, we hold the belief that for FFPE specimens undergoing lab-based XRH imaging, radiation damage is not a significant concern. From internal source characterisation experiments (data not shown) we have measured that for XRH relevant beam parameters (80kVp, 8W, 50 mm source to object distance) we get a Dose rate of c. 30 mGy/s; that is a deposited dose of c. 325 Gy for a 3h scan. Of course, the actual number would differ with filtration, distance from the source, embedding medium, scan duration etc, but they will remain in the same order of magnitude. E.F. Garmana & M. Weik suggest 58 kGy is a low dose (diffraction weighted) while structural studies done using enzymes show structural damage occurring between 0.13 -3.88 MGy (Taberman et al., 2019 in the same); that is 3 to 4 orders of magnitude higher.  Given the steadily increasing X-ray flux densities available at Synchrotron facilities, coupled with the practical constraints that often limit scan duration in lab-based XRH (such as cost, availability, and throughput), it's reasonable to assert that tissue studies utilising SRμCT typically entail significantly higher radiation doses. It's worth noting that this doesn't imply that laboratory-based μCT cannot reach these levels of exposure or that SRμCT cannot be optimised for low-dose imaging.   What's particularly noteworthy, especially for conventional histology applications, is that Teplov et al. offer initial evidence suggesting that lab-based XRH does not impact protein expression and, consequently, the affinity of tissues for histological stains. Taking into consideration these comments we will be revising the relevant part as follows:

- “ *Another point of consideration that should be addressed when discussing experimental planning with the client is the prospect of radiation-induced specimen damage. Such damage has the potential to influence subsequent biochemical analyses, including immunohistochemistry, proteomics, and transcriptomics. Notably, existing research in this area has predominantly focused on synchrotron radiation-based studies, where substantial doses have been found to impact tertiary protein structures [54]. Nevertheless, for laboratory-based radiation doses, especially within the context of the low-dose protocols typically used for soft tissue imaging in our facility (0.1 -5.0 kGy), there appears to be minimal impact on the tissue, especially concerning the affinity of tissues for histological stains [7].”*
